# Structure and Multitasking of the c-di-GMP-Sensing Cellulose Secretion Regulator BcsE

**DOI:** 10.1128/mBio.01303-20

**Published:** 2020-08-11

**Authors:** Samira Zouhir, Wiem Abidi, Meryem Caleechurn, Petya Violinova Krasteva

**Affiliations:** a Structural Biology of Biofilms Group, Institute for Integrative Biology of the Cell (I2BC), CEA, CNRS, Paris-Sud University, Gif-sur-Yvette, France; b Structural Biology of Biofilms Group, European Institute of Chemistry and Biology (IECB), Pessac, France; c CBMN UMR 5248 CNRS, University of Bordeaux, Pessac, France; University of Washington

**Keywords:** biofilm formation, c-di-GMP signaling, cellulose secretion, structural biology

## Abstract

Most bacteria respond to surfaces by biogenesis of intracellular c-di-GMP, which inhibits motility and induces secretion of biofilm-promoting adherence factors. Bacterial cellulose is a widespread biofilm component whose secretion in Gram-negative species requires an inner membrane, c-di-GMP-dependent synthase tandem (BcsAB), an outer membrane porin (BcsC), and various accessory subunits that regulate synthase assembly and function as well as the exopolysaccharide’s chemical composition and mechanical properties. We recently showed that in Escherichia coli, most Bcs proteins form a megadalton-sized secretory nanomachine, but the role and structure of individual regulatory components remained enigmatic. Here, we demonstrate that essential-for-secretion BcsR and BcsQ regulate each other’s folding and stability and are recruited to the inner membrane via c-di-GMP-sensing BcsE and its intraoperon partner BcsF. Crystallographic and solution-based data show that BcsE’s predicted GIL domain is a degenerate receiver-GGDEF domain tandem (BcsE^REC^*^-GGDEF^*), where the divergent diguanylate cyclase module binds both dimeric c-di-GMP and BcsQ through mutually independent interfaces. In addition, we reveal that a third N-terminal domain (BcsE^NTD^) determines the protein’s homooligomerization and targeting of BcsERQ to the membrane as well as previously unreported interactions with transcription antitermination complex components. Together, the data suggest that BcsE acts on multiple levels to fine-tune bacterial cellulose secretion, from the early stages of secretion system assembly to the maintenance of a membrane-proximal pool of dimeric c-di-GMP for processive synthase activation.

## Abstract (precis)

Bacterial cellulose is a widespread biofilm component that can modulate microbial fitness and virulence both in the environment and infected hosts. Whereas its secretion generally involves an inner membrane c-di-GMP-dependent synthase tandem (BcsAB) across the bacterial domain of life, enterobacteria feature sophisticated Escherichia coli-like Bcs secretion systems, where multiple additional subunits are either required for secretion or contribute to the maximal production of the polysaccharide *in vivo.* Here, we demonstrate that essential-for-secretion BcsR and BcsQ regulate each other's folding and stability and are recruited to the inner membrane via c-di-GMP-sensing BcsE and its intraoperon partner, BcsF. Crystallographic and functional data reveal that BcsE features unexpected domain architecture and likely acts on multiple levels to fine-tune bacterial cellulose production, from the early stages of secretion system assembly to the maintenence of a membrane-proximal pool of dimeric c-di-GMP for processive synthase activation.

## INTRODUCTION

Bacterial biofilm formation is a ubiquitous adaptational strategy that provides fitness and resistance advantages to both free-living and clinically important species (1). In most motile bacteria, the switch from planktonic to biofilm life styles is orchestrated by an intracellular second messenger, c-di-GMP, that acts at the transcriptional, translational and posttranslational levels to inhibit flagellar motility and induce the secretion of extracellular matrix components (2, 3). Bacterial cellulose is a widespread biofilm exopolysaccharide that typically requires an inner membrane, c-di-GMP-dependent synthase tandem for glucose polymerization and inner membrane transport (BcsAB), and in Gram-negative species, an outer membrane porin with peptidoglycan-binding scaffolding motifs (BcsC) (4). Depending on the type of core and accessory subunits, four major types of cellulose secretion systems are generally recognized among bacteria (5). Many *Betaproteobacteria* and *Gammaproteobacteria* feature sophisticated Escherichia coli-like systems for cellulose biogenesis, where multiple additional subunits are either essential for secretion or contribute to the maximal production of the polysaccharide *in vivo* (5, 6).

**FIG 1:**
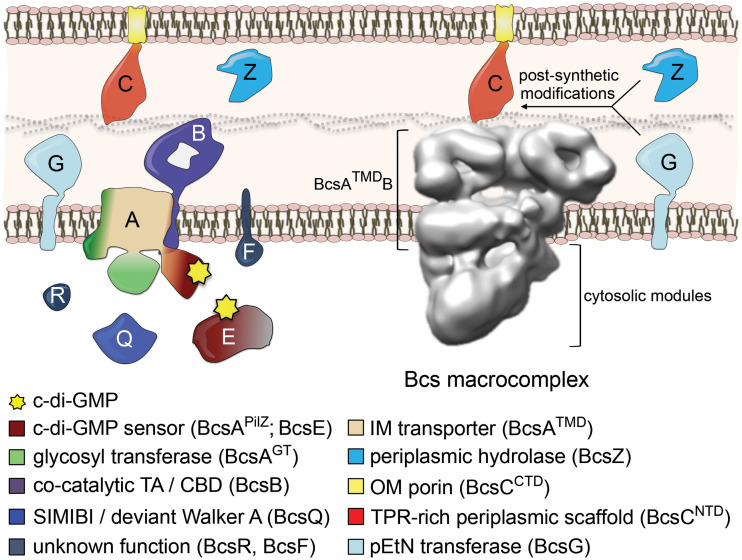
E. coli-like cellulose secretion systems. (Left) Thumbnail representation and proposed topology of the nine Bcs proteins. (Right) Electron microscopy-based three-dimensional reconstruction of the Bcs macrocomplex, encompassing most of the inner membrane and cytosolic subunits. Known and proposed roles for the different subunits and/or protein domains are color coded and annotated at the bottom. GT, glycosyl transferase domain; TA, tail-anchor; CBD, carbohydrate-binding domains; SIMIBI, signal recognition particle, MinD and BioD superfamily; TMD, transmembrane domain(s); IM, inner membrane; OM, outer membrane; NTD, N-terminal domain; CTD, C-terminal domain; TPR, tetratricopeptide repeats; pEtN, phosphoethanolamine.

In particular, the E. coli *bcsEFG* and *bcsRQABZC* operons encode a total of nine subunits that span from the cytosol to the surface of the cell (5, 6) (Fig. 1). The processive glucose polymerization reaction is carried out by a glycosyl transferase domain on BcsA (BcsA^GT^), whose active site is made accessible by the recurrent binding of dimeric intercalated c-di-GMP to an adjacent PilZ β-barrel domain on the protein (BcsA^PilZ^) and the displacement of a so-called gating loop capping the substrate-binding pocket (7, 8). Transport is coupled to polymerization, and the nascent polysaccharide chain is extruded, one molecule at a time, through the inner membrane transport domain of BcsA completed by the C-terminal tail-anchor of the cocatalytic subunit BcsB (BcsB^TA^) (4, 7). We showed earlier that in E. coli, most of the inner membrane and cytosolic Bcs components interact stably to form a megadalton-sized Bcs macrocomplex with a seashell-like, layered, and asymmetric architecture (Fig. 1) (6). In it, multiple copies of BcsB arrange in a fan-like assembly, or “crown,” in the periplasm, which is proposed to lead the outcoming cellulose toward the outer membrane secretory component BcsC (6). En route, the synthesized cellulose can undergo enzymatic modifications through the addition of phosphoethanolamine residues by BcsG or limited hydrolysis by BcsZ (4, 5, 9).

Interestingly, E. coli-like cellulose secretion *in vivo* is absolutely dependent on the expression of two small cytosolic proteins, BcsR and BcsQ (6), whose genes precede those for the membrane-embedded secretory components in their respective *bcs* operon. We showed earlier that deletion of the BcsB periplasmic modules (BcsB^peri^) did not abolish Bcs macrocomplex assembly (6), indicating that membrane targeting of the cytosolic components likely precedes the multimerization of BcsB protomers in the crown. BcsR is a short 7-kDa polypeptide with unknown structure and function, whereas BcsQ is predicted to belong to the ancient SIMIBI (signal recognition particle, MinD and BioD) superfamily of NTPases (10, 11). Members of the latter are key to a large variety of cellular processes, including bacterial flagellar secretion (FlhG and FlhF) and membrane protein sorting in both prokaryotes and higher organisms (SRP54-SR and Get3) (10, 11). This, together with our earlier observations that BcsQ affects detection of the downstream BcsA synthase in the membrane (6), suggests that BcsQ might play a role in the early stages of cellulose secretion system assembly. A third cytosolic protein, BcsE, has been shown to significantly boost cellulose secretion *in vivo* and to present a second c-di-GMP binding module in addition to the BcsA^PilZ^ domain (6, 12). Previous work has defined BcsE as a GGDEF-I-site-like (GIL) domain-containing protein due to c-di-GMP recognition by a conserved RXXD sequence, which, when found on diguanylate cyclases, can serve as a product-sensing regulatory motif called “I-site” (12). Finally, the Bcs secretion system is completed by a short membrane-embedded polypeptide, BcsF, that is also necessary for maximal cellulose production *in vivo* through an as-yet-unknown mechanism (6).

We showed earlier that both BcsF and the cytosolic BcsERQ components assemble stably with the inner membrane BcsAB biosynthetic platform to form the seashell-like Bcs macrocomplex visualized by single-particle electron microscopy (Fig. 1) (6). The low resolution of these structural data, however, precluded us from gaining specific insights into individual regulatory components or the interdependence of Bcs subunit interactions. Here, we determine that essential-for-secretion BcsR and BcsQ determine each other’s folding and stability and that their membrane targeting is facilitated by high-affinity interactions with the c-di-GMP sensor BcsE. To unravel the latter’s structure and function, we solved the crystal structure of a stable, N-terminally truncated BcsE variant (BcsE^217−523^) and reveal that the previously postulated GIL domain is in fact a degenerate receiver-GGDEF domain tandem (REC*-GGDEF*). We further show that the catalytically incompetent diguanylate cyclase module senses through separate interfaces both BcsQ and c-di-GMP and that the dinucleotide likely adopts a dimeric conformation in solution, such as the one necessary for processive BcsA gating loop displacement and glucose polymerization (8). We also present evidence that although BcsQ is recruited by the C-terminal BcsE^GGDEF^* domain, efficient BcsERQ membrane targeting requires the remaining N-terminal module (BcsE^NTD^) and that membrane partitioning is largely triggered by inner membrane BcsF. Finally, we determine that BcsE further uses its N-terminal domain to both homooligomerize and interact with transcription antitermination complex (TAC) components and discuss a putative physiological role for these unexpected interactions. Together, the data presented here suggest that BcsE and BcsF proteins might have evolved in E. coli-like cellulose secretion systems to boost exopolysaccharide production through actions at multiple levels: from high-affinity sequestration and membrane targeting of essential-for-secretion components to the maintenance of a membrane-proximal pool of dimeric c-di-GMP for processive synthase activation.

## RESULTS AND DISCUSSION

### BcsR and BcsQ interdependence and heterocomplex formation.

**FIG 2:**
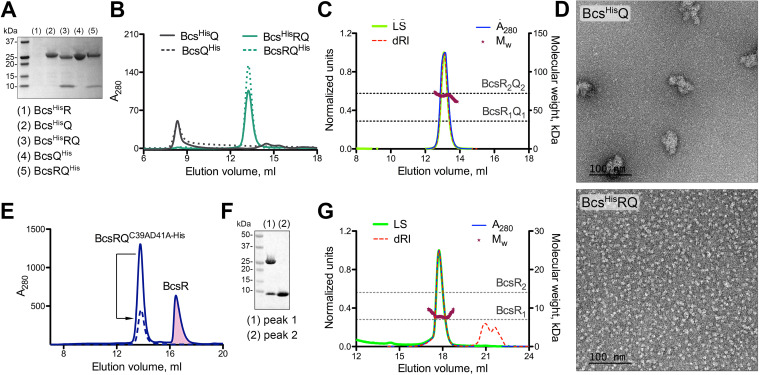
BcsR and BcsQ interdependence and complex formation. (A) IMAC elution fractions upon expression of Bcs^His^R (pProExHTB-Bcs^His^R), Bcs^His^Q (pProExHTB-Bcs^His^Q), Bcs^His^RQ (pProExHTB-Bcs^His^RQ), BcsQ^His^ (pET21b-BcsQ^His^), and BcsRQ^His^ (pET21b-BcsRQ^His^). (B) Size exclusion chromatography (SEC) profiles of the purified proteins from panel A (2 to 5) using a Superdex 200 Increase 10/30 GL column. (C) SEC-coupled multiangle light scattering (SEC-MALS) of purified tag-free BcsRQ complex. Normalized experimental traces for the light scattering (LS), differential refractive index (dRI), UV absorbance at 280 nm (*A*_280_) and calculated molecular weight (*M*_w_) are annotated at the top, theoretical molecular weights for the BcsRQ complex at 1:1 and 2:2 stoichiometries are shown as dashed lines. (D) Electron micrographs in negative stain of the purified Bcs^His^Q (top) and Bcs^His^RQ complex (bottom). (E) Purification of folded noncomplexed BcsR upon coexpression with the BcsQ^C39AD41A-His^ mutant following IMAC and SEC. The BcsR peak is colored in pink. A dashed line shows the SEC profile of the mutant BcsRQ^C39AD41A-His^ complex upon reinjection. (F) SDS-PAGE analysis of fractions corresponding to the two peaks in panel E. (G) SEC-MALS of the purified BcsR protein with experimental and theoretical traces as described above.

In cellulose-producing enterobacteria, *bcs* genes are typically arranged in two separate operons, with hallmark *bcsRQ* and *bcsE* genes featuring promoter-proximal positions in each (5, 6). Different efforts to purify BcsR and BcsQ constructs on their own were not successful, with the proteins failing to express stably (Bcs^His^R) or aggregating upon purification (Bcs^His^Q and BcsQ^His^) (Fig. 2; see also Table S1 in the supplemental material). Coexpression of the two subunits, however, led to the stable expression and purification of a homogeneous heterotetrameric BcsRQ complex with apparent 2:2 stoichiometry in solution, where BcsR-dependent BcsQ stabilization appeared independent of the presence or position of epitope tags on the subunits (Bcs^His^RQ, BcsRQ^His^, and BcsRQ) (Fig. 2A to D). Interestingly, while individual expression of BcsR did not yield detectable levels of purified protein, we identified empirically BcsQ variants that, when coexpressed under the same promoter with BcsR (BcsRQ^C39AD41A-His^ and BcsRQ^C39AD41AL43D-His^), yielded an excess of purified BcsR protein, which remained relatively stable in monomeric form in solution (Fig. 2E to G). These data indicate that the two proteins likely exhibit chaperone-like functions toward each other, where BcsR stabilizes BcsQ to form monodisperse heterotetramers in solution, while BcsQ itself might play a role in the folding and subsequent stability of BcsR.

10.1128/mBio.01303-20.4 TABLE S1 Protein expression constructs used in the study. Download Table S1, PDF file, 0.1 MB. Copyright © 2020 Zouhir et al. 2020 Zouhir et al. This content is distributed under the terms of the Creative Commons Attribution 4.0 International license.

Although bacterial operons have now been described for more than half a century (13), only recently have mechanistic insights into the role of operon organization begun to emerge. In particular, not only are proteins that function together through the assembly of heteromeric complexes likely to be encoded by genes in the same or adjacent operons, but operon gene order has also been reported as generally optimized for the order of protein complex assembly itself (14). This appears to be especially true for low-copy systems, as are typically the energetically costly secretion systems, where expression-coupled protein-protein interactions would minimize the stochasticity of heterocomplex formation (14). Nevertheless, protein folding in the context of multiprotein assemblies, as well as intraoperon partners remains enigmatic. Studies on native and engineered proteins have shown that charged or intrinsically disordered N-terminal domains and protein tails can act as so-called “entropic bristles” with protein folding helper effects that stabilize fused downstream modules by minimizing their intrinsic aggregation propensity (15–17). We propose here that BcsRQ complex formation represents a paradigm of similar folding helper effects at the intraoperon level where upstream expression of an initially disordered BcsR minimizes the aggregation of its intraoperon partner BcsQ. The sequential expression of the two proteins could therefore not only limit the stochasticity of complex assembly within a low-copy cellulose secretion system but also couple the inhibition of intermolecular BcsQ aggregation with the intramolecular folding of BcsR to secure a stable stoichiometric assembly. Moreover, maintenance of separate polypeptides versus the evolution of genetically fused modules could present further advantages of operon organization, such as additional regulatory inputs or possible stoichiometry and symmetry variations upon secretion system assembly and function. In support for this model, recent work from our group has revealed that even in the context of the stable BcsR_2_Q_2_ heterocomplex, BcsR features a highly flexible and partly disordered N-terminal region that can partake in nonsymmetric protein-protein interactions, whereas the C-terminal domain adopts an α-helical fold at the interface of two BcsQ protomers (W. Abidi, S. Zouhir, M. Caleechurn, S. Roche, and P. V. Krasteva, unpublished).

### BcsERQ complex formation and membrane targeting.

**FIG 3:**
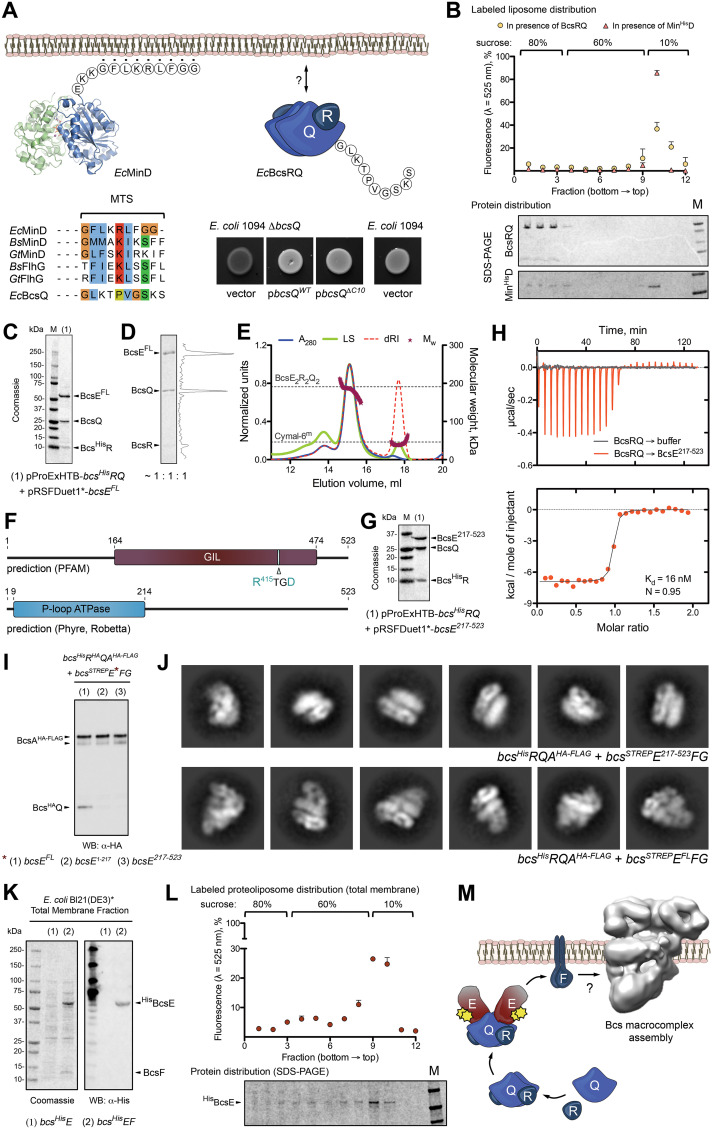
BcsRQ membrane targeting and BcsERQ complex formation. (A) Testing the presence and role of a putative membrane targeting sequence (MTS). (Top left) crystal structure and MTS sequence of the E. coli MinD protein. (Bottom left) MTS conservation among representative SIMIBI proteins and comparison with the corresponding BcsQ C-terminal tail (C10). (Top right) Thumbnail representation of the BcsRQ complex and C10 sequence. (Bottom right) Calcofluor binding assay for cellulose secretion in wild-type (positive control) and mutant (Δ*bcsQ*) E. coli 1094 upon complementation with wild-type or C10-truncated BcsQ. Transformation with an empty pAM238 vector in the Δ*bcsQ* background was used as a negative control. (B) Liposome flotation assay of potential BcsRQ-lipid interactions. (Top) Relative fluorescence of sucrose gradient fractions after NBD-PE-labeled liposome flotation in the presence of Min^His^D (positive control) or BcsRQ. (Bottom) Representative SDS-PAGE analysis of protein distribution along the gradient fractions. Migrated proteins were stained with Coomassie. (C) SDS-PAGE analysis of the IMAC elution fraction upon coexpression of a Bcs^His^R, BcsQ, and BcsE^FL^ complex (pProExHTB-Bcs^His^RQ plus pRSFDuet1*-BcsE^FL^ coexpression strategy). (D) Calculated protein ratio in the purified tag-free BcsERQ^FL^ complex based on densitometric analysis of the SDS-PAGE migrated bands. (E) SEC-MALS of the purified BcsERQ^FL^ complex. Experimental and theoretical traces (as described above) are shown for both protein and detergent micelle (Cymal-6^m^) peaks. (F) Conserved domain detection using sequence alignment and fold prediction tools. (G) SDS-PAGE analysis of the IMAC elution fraction of upon coexpression of a Bcs^His^R, BcsQ, and BcsE^217−523^ complex (pProExHTB-Bcs^His^RQ plus pRSFDuet1*-BcsE^217-523^ coexpression strategy). (H) Isothermal titration calorimetry (ITC) profile of the BcsE^217−523^ → BcsRQ interaction. (I) Western blot analysis of the Bcs^HA^Q integration into anti-FLAG tag-purified Bcs macrocomplex (Bcs^His^R^HA^QA^HA-FLAG^B-^STREP^EFG coexpression) in the context of BcsE^FL^, BcsE^1−217^, and BcsE^217−523^. (J) Representative views (class averages) of Bcs macrocomplex carrying BcsE^217−523^ (top) versus BcsE^FL^ (bottom, control). (K) BcsE^FL^ membrane targeting in the context of BcsF coexpression (pProExHTB-Bcs^His^E^FL^ versus pProExHTB-Bcs^His^E^FL^F expression strategies). (Left) SDS-PAGE analysis of the total membrane fractions; (right) Western blot detection of Bcs^His^E^FL^ in the corresponding fractions. (L) Liposome flotation experiments using NBD-PE-labeled total membrane proteoliposomes from cells coexpressing Bcs^His^E^FL^ and BcsF (pProExHTB-Bcs^His^E^FL^F coexpression). (Top) Relative fluorescence of the gradient fractions indicating proteoliposome distribution; (bottom) SDS-PAGE analysis of Bcs^His^E^FL^ distribution across the corresponding gradient fractions. (M) Results summary showing proposed membrane targeting and macrocomplex integration of the essential for secretion subunits BscR and BcsQ via cytosolic BcsE and membrane-embedded BcsF.

We previously showed that although predicted as hydrophilic cytosolic proteins, BcsRQ associate stably with pelleted membranes in cell fractionation experiments and subsequently copurify with the detergent-extracted Bcs macrocomplex (6). Based on sequence conservation and putative fold recognition, BcsQ belongs to the ancient family of SIMIBI NTPases, many of which are involved in membrane-mediated processes such as division septum inhibition (MinD), flagellar assembly (FlhG and FlhF), protein secretion, and membrane protein sorting (Srp54-SR and Get3), among others (10, 11). While some of these proteins are targeted to the membrane via specific protein-protein interactions, others, such as MinD and FlhG homologs, have intrinsic membrane-targeting sequences (MTS) that are proposed to adopt an amphipathic α-helical fold upon contact with membrane lipids (Fig. 3A) (18–20). Comparative sequence analysis shows that a conserved basic residue midway in the MTSs of MinD and FlhG homologs is replaced by a proline in the corresponding 10-residue-long C-terminal tail of BcsQ (BcsQ^C10^) (Fig. 3A). Although proline is generally a potent breaker of both α-helical and β-strand secondary structures in aqueous environments, it is often found in putative transmembrane protein helices and has been shown to protect α-helical conformations in hydrophobic milieus (21). To determine the potential role of BcsQ^C10^ in membrane targeting, we performed cell-based phenotypic and *in vitro* lipid-binding assays. Interestingly, deletion of the BcsQ^C10^ region had no significant effect on cellulose secretion in a functional complementation assay *in vivo* (Fig. 3A), and purified BcsRQ failed to partition with the lipid-enriched fractions in liposome flotation experiments *in vitro* (Fig. 3B). As these data favor protein-based membrane targeting of the essential-for-secretion BcsRQ complex, we proceeded to determine the nature and sequence of downstream BcsRQ interactions. We started by probing putative interactions with the third cytosolic component, BcsE, and after testing different strategies for BcsERQ recombinant coexpression (see Materials and Methods), we were able to purify a stable BcsERQ heterocomplex with equimolar 2:2:2 stoichiometry in solution (Fig. 3C to E).

BcsE occupies a leader position in its respective *bcsEFG* operon, which is consistent with a role in the early stages of Bcs macrocomplex assembly (14). Residues 164 to 474 of the protein were previously defined as a conserved GGDEF I-site-like (GIL) domain based on the identification of a c-di-GMP binding RXXD motif, similar to the I-site regulatory sequence often found on diguanylate cyclases (12) (Fig. 3F). Interestingly, fold recognition programs predict that the N-terminal BcsE region, which features significantly lower overall sequence conservation (see Fig. S1A), adopts a RecA-like ATPase fold whose boundaries significantly overlap those of the postulated C-terminal GIL domain of the protein (Fig. 3F) (22, 23). To identify stable BcsE modules and examine their role in secretion system assembly, we used both sequence conservation criteria and predicted three-dimensional fold models to create a series of N- and C-terminally truncated BcsE variants for recombinant coexpression. From these, we identified a construct, BcsE^217−523^, that copurifies with BcsRQ similarly as full-length BcsE (Fig. 3G). When individually expressed, the truncated variant featured higher purity, stability, and protein yields than BcsE^FL^, which allowed us to obtain a thermodynamic profile of the BcsRQ → BcsE interaction and reveal a dissociation constant in the low nanomolar range (*K_d_* ≈ 16 nM) (Fig. 3H). Considering that the binding affinity is likely even higher in the crowded high-viscosity environment of the bacterial cytosol and that the typical volume of an E. coli cell is in the low femtoliter range (24), these data indicate that as soon as the first copies of folded BcsRQ heterocomplex are formed, they will be bound and sequestered by their BcsE partners *in vivo*.

10.1128/mBio.01303-20.1 FIG S1 Enterobacterial BcsE. (A) Sequence conservation of BcsE^FL^ in representative enterobacterial species. Blue color intensity/darkness correlates with sequence conservation. Putative and observed domains as well as resolved secondary structure elements are annotated. c-di-GMP (CDG) coordinating residues are marked with white triangles. The alignment was generated in Clustal Ω and visualized in Jalview. (B) BcsE^217−523^ surface conservation mapping. (Left) Surface representation of the resolved crystal structure with the REC* domain colored in orange, the linker in black, the interstitial helix in beige, the GGDEF* domain in dark red, the c-di-GMP coordinating residues in cyan, and the divergent GGDEF loop in blue. c-di-GMP is represented in sticks. Images were generated in PyMol. (Right) Enterobacterial surface conservation mapped onto the respective orientations with conservation scores represented as a cyan (0%) to maroon (100%) color gradient. Images were generated in UCSF Chimera. Download FIG S1, PDF file, 2.8 MB. Copyright © 2020 Zouhir et al. 2020 Zouhir et al. This content is distributed under the terms of the Creative Commons Attribution 4.0 International license.

Interestingly, biochemical and electron microscopy data show that Bcs complexes purified via a C-terminal FLAG tag on BcsA fail to efficiently incorporate cytosolic Bcs components upon deletion of either the N-terminal BcsE^1−217^ or the C-terminal BcsE^217−523^ regions (Fig. 3I and J). While the latter can be explained by disrupted BcsERQ complex formation through deletion of the BcsRQ binding module, the effects of BcsE^1−217^ deletion indicate that this N-terminal domain remains virtually indispensable for BcsERQ membrane targeting and its stable incorporation into the native Bcs macrocomplex.

We previously showed that deletion of BcsE intraoperon partners BcsF and BcsG have similar effects of incomplete macrocomplex assembly as the deletion of BcsE^1−217^ shown here (Fig. 3J) (6). Though both BcsF and BcsG are inner membrane proteins, BcsG is involved in covalent modifications of the secreted cellulose in the periplasm and does not purify stably with the assembled Bcs macrocomplex (6, 9). We therefore hypothesized that of the two, BcsF is more likely to act at the early stages of Bcs macrocomplex assembly as a membrane triggering factor. To test this, we examined BcsE membrane partitioning in the presence or absence of BcsF (Bcs^His^E^FL^ versus Bcs^His^E^FL^F expression). Indeed, BcsEF coexpression led to enrichment of BcsE in the pelleted total membrane fraction (Fig. 3K), and the protein partitioned with membrane-derived proteoliposomes upon flotation, effectively ruling out potential aggregation in the coexpression context (Fig. 3L). These data provide further support for coordinated subunit expression and protein complex assembly, where BcsRQ-bound BcsE is subsequently recruited to the inner membrane by its immediate downstream operon neighbor, BcsF.

Interestingly, in Pseudomonas putida, the BcsF gene is preceded by two putative open reading frames (PP_2629 and PP_2630), each of which shares conservation with the BcsE^1−217^ or Bcs^217−523^ fragments empirically characterized here (see Fig. S2A and B). This, together with the significant difference in sequence conservation between the two BcsE fragments, points toward the evolution of multidomain enterobacterial BcsE from the genetic fusion of smaller protein subunits to secure not only c-di-GMP recognition as known for the so-called I-site RXXD motif but also efficient BcsRQ complexation and subsequent delivery to the inner membrane biosynthetic platform via high-affinity BcsE-BcsF interactions (Fig. 3M).

10.1128/mBio.01303-20.2 FIG S2 Sequence conservation of Pseudomonas putida PP_2629 (A) and PP_2630 (B) compared to the enterobacterial BcsE consensus sequence derived from the alignment in Fig. S1. X indicates a nonconserved, or any, amino-acid. Download FIG S2, PDF file, 2.3 MB. Copyright © 2020 Zouhir et al. 2020 Zouhir et al. This content is distributed under the terms of the Creative Commons Attribution 4.0 International license.

### Crystal structure of BcsE^217−523^.

**FIG 4:**
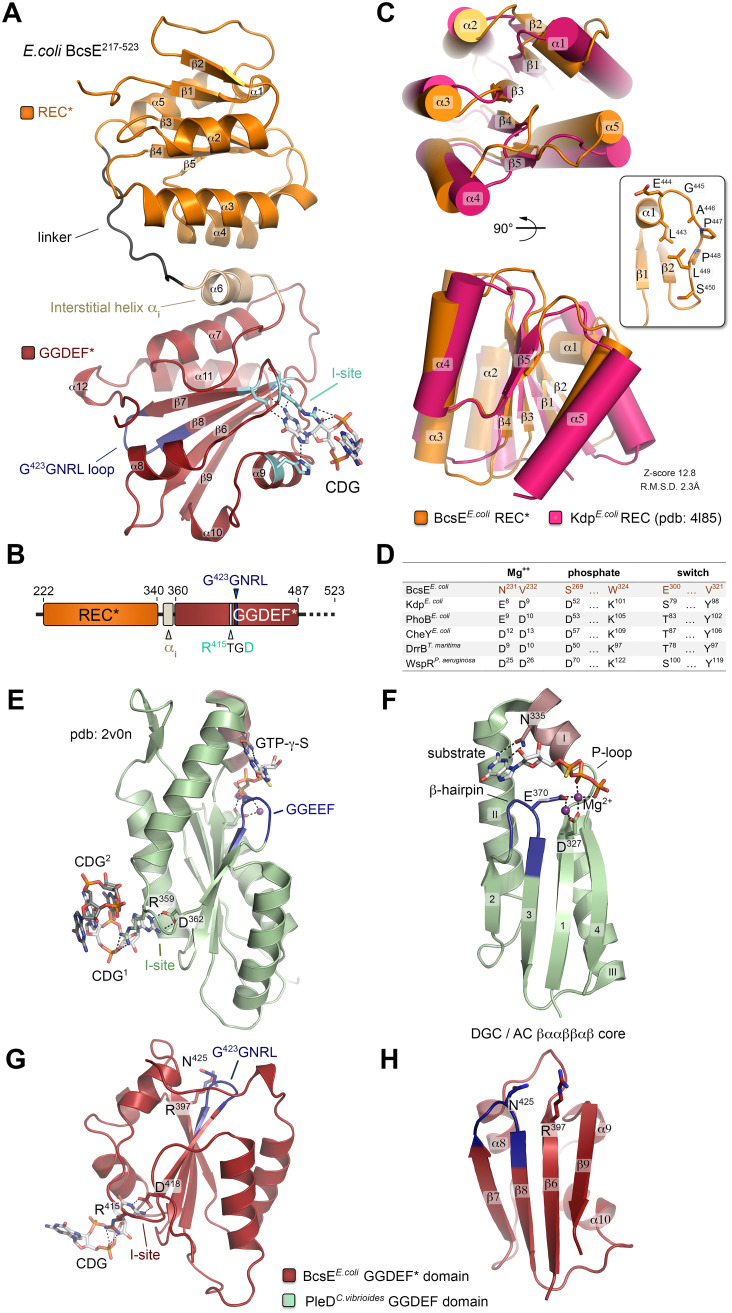
BcsE^217−523^ crystal structure and domain organization. (A) Crystal structure of BcsE^217−523^. The degenerate receiver (REC*) and diguanylate cyclase (GGDEF*) domains are colored orange and red, respectively, and key motifs are highlighted. Secondary structure elements are numbered without accounting for the missing N-terminal domain. (B) Summary of the resolved domain architecture for the previously predicted GIL domain. (C) Overlay of the E. coli BcsE^REC^* domain and a canonical receiver domain (E. coli Kdp^REC^) in two different views. Inset, unfolding of the canonical α1 helix into a P-rich loop. Structural alignment scores are calculated in DALI. (D) Comparison of key conserved residues in phosphotransfer-competent response regulators with corresponding residues in BcsE^REC^*. (E) GGDEF domain, I-site-mediated c-di-GMP binding, and substrate homologue coordination of the catalytically active diguanylate cyclase PleD*^C. vibiroides^*. (F) Conserved βααββαβ catalytic core shared among adenylate and diguanylate cyclases. Key residues involved in substrate and Mg^2+^ coordination are shown as sticks. Crystal structures of the corresponding BcsE^GGDEF^* domain (G) and α/β core (H).

**FIG 5:**
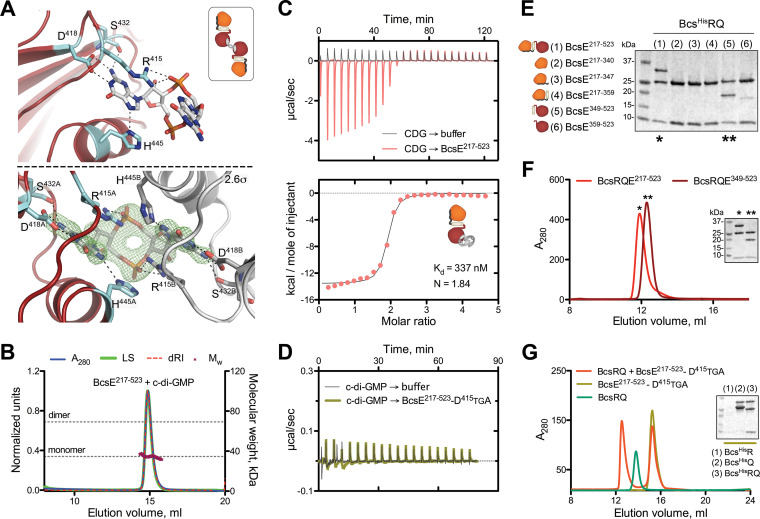
c-di-GMP binding and BcsE-BcsQ interactions. (A) c-di-GMP binding to the conserved I-site. (Top) Stick representation of c-di-GMP and the coordinating residues with only one protein molecule shown. (Inset) Thumbnail representation of the 2:1 protein-to-dinucleotide complexation observed in the crystals. (Bottom) An (|Fo|-|Fc|) partial electron density map calculated from a model prior to inclusion of the dinucleotide and contoured at 2.6σ with both coordinating protomers shown in red/cyan and gray. (B) SEC-MALS of BcsE^217−523^ in the presence of excess c-di-GMP with experimental and theoretical traces as described above. (C) ITC profile of the c-di-GMP → BcsE^217−523^ interaction and thumbnail representation of the calculated binding stoichiometry (1:2, protein to dinucleotide). (D) Control ITC titration of c-di-GMP to the I-site-defective BcsE^217−523^-D^415^TGA mutant. (E) SDS-PAGE analysis of IMAC elution fractions testing BcsERQ complex formation upon Bcs^His^RQ coexpression with various truncated BcsE variants (pProExHTB-Bcs^His^RQ plus pRSFDuet1*-BcsE^trunc^ coexpression). (F) SEC profiles of the purified BcsERQ^GGDEF^* complex and BcsERQ^217−523^. (G) SEC profile of purified BcsRQ preincubated with excess BcsE^217−523^-D^415^TGA compared to profiles for separate injections of the individual components. (Inset) SDS-PAGE analysis of IMAC elution fractions assaying BcsE^217−523^-D^415^TGA copurification upon coexpression with Bcs^His^R, Bcs^His^Q, or Bcs^His^RQ.

To gain further insights into BcsE structure and function, we pursued crystallization of the stable C-terminal BcsE^217−523^ construct, which encompasses most of the postulated GIL domain module. Purified untagged BcsE^217−523^ crystallized in the presence of c-di-GMP, and its structure was determined to 2.2 Å using single-wavelength anomalous dispersion (SAD) phasing on crystals grown from selenomethionine-derivatized protein (Table S2). The protein packed in the P4_1_2_1_2 space group with two BcsE^217−523^ molecules per asymmetric unit adopting virtually identical conformations with root mean square deviation (RMSD) of 0.835 Å over all atoms. A single c-di-GMP is found splayed in a symmetrical conformation between the two protomers, and nucleotide recognition involves four residues of each subunit, namely, R^415^ and D^418^ from the conserved I site-like motif as well as the side chain of H^445^ and the peptide carbonyl of S^432^ (Fig. 4 and 5). Unexpectedly, the construct adopts a dual-domain fold with an apparent N-proximal module connecting via an interstitial helix (α_6_) to a C-terminal domain, in which the last ∼40 residues (C-terminal tail) remain unresolved in the structure (Fig. 4A and B).

10.1128/mBio.01303-20.5 TABLE S2 Crystallographic data collection and refinement statistics. Download Table S2, PDF file, 0.1 MB. Copyright © 2020 Zouhir et al. 2020 Zouhir et al. This content is distributed under the terms of the Creative Commons Attribution 4.0 International license.

A search for three-dimensional (3D) structural homologs using the fold recognition server DALI (25) revealed that the N-proximal domain adopts a receiver (REC) domain-like (βα)_5_ fold (26), where the central five-stranded parallel β-sheet is flanked by 4 α-helices, while the canonical α_1_ is mostly unfolded in an extended conformation by a stretch of proline and other small uncharged amino acids (Fig. 4C). Canonical REC domains are typically found in tandem with DNA-binding modules in response regulator proteins, which use phosphoryl transfer from upstream kinases as input signals for transcription regulation (26). Structural and sequence alignments of BcsE^REC^* with phosphorylation-competent receiver domains, however, show significant deviation from the amino acid consensus of key functional motifs, indicating that the module is unlikely to function in phosphotransfer-dependent signal transduction (Fig. 4D).

Similar DALI search using the resolved C-proximal domain as an input revealed the closest structural homolog as the cytosolic C-terminal domain of Pseudomonas aeruginosa PelD. Interestingly, the latter is itself a c-di-GMP-binding protein responsible for the activation of synthase-dependent exopolysaccharide secretion, the Pel system in pseudomonads, and has been characterized as a degenerate GGDEF domain-containing protein where c-di-GMP sensing is carried out by the conserved I-site motif (27–29). Indeed, both BcsE^GGDEF^* and PelD^GGDEF^* show severe degeneration of the consensus βααββαβ catalytic core shared by diguanylate and adenylate cyclases (30), with the substrate-coordinating P-loop and α_1_-helix completely missing and catalytic residues, including those from the signature GGDEF motif, showing significant divergence (Fig. 4E to H; see also Fig. S3). Nevertheless, I-site-dependent c-di-GMP complexation remains virtually unchanged from that of active diguanylate cyclases, with the dinucleotide participating in both polar and π-stacking interactions with the side chains of the conserved arginine (R^415^) and aspartate (D^418^) residues (Fig. 4E to H and 5A; Fig. S3). Taken together, these results classify BcsE as a member of a growing superfamily of c-di-GMP-sensing proteins, in which canonical signaling (REC, PAS, etc.) or enzymatic (GGDEF, EAL, etc.) modules have been repurposed to serve c-di-GMP-dependent signal transduction (2, 3).

10.1128/mBio.01303-20.3 FIG S3 c-di-GMP complexation. BcsE^217−523^-c-di-GMP structural models based on crystallographic (A) versus solution-based (B) data. Published crystal structures of the closest structural homolog, P. aeruginosa PelD^GGDEF^* showing coordination of a single (C) or two intercalated (D) c-di-GMP molecule(s). The structural similarity search was performed using the DALI server. (E and F) c-di-GMP-dependent active site opening for UDP recycling and UDP-glucose entry in Rhodobacter sphaeroides BcsA. Gating loop residues are shown in dark blue, the glycosyl transferase domain (BcsA^GT^) in green, the inner membrane domain in beige (BcsA^TMD^), and the PilZ domain (BcsA^PilZ^) and adjoining linkers in dark red. UDP is shown as spheres and side chains, cellulose, and dimeric c-di-GMP are shown as sticks. Illustrations were generated in PyMol. Download FIG S3, PDF file, 2.8 MB. Copyright © 2020 Zouhir et al. 2020 Zouhir et al. This content is distributed under the terms of the Creative Commons Attribution 4.0 International license.

### C-di-GMP and BcsQ binding by the BcsE^GGDEF^* domain.

Although the primary sequence of the resolved BcsE^REC^*^-GGDEF^* modules is overall highly conserved, surface mapping of the amino acid conservation reveals distinct conserved residue clusters on both the degenerate receiver and diguanylate cyclase modules, which might be indicative of oligomerization or protein-protein interaction interfaces (Fig. S1). To determine the protein’s homooligomerization propensity in solution, we performed solution-based light-scattering experiments and determined that the BcsE^217−523^ construct remains monomeric even in the presence of saturating c-di-GMP (Fig. 5B). This is particularly surprising considering the intrinsic dimerization propensity of REC domains in general (26), the binding stoichiometry of the BcsE^217−523^RQ complex in solution (N ≈ 0.95, consistent with 2:2:2 binding) (Fig. 3H), and the symmetrical c-di-GMP conformation in the crystal structure (Fig. 5A, bottom and inset; Fig. S3A and B), where the dinucleotide bridges two separate BcsE protomers by identical interactions. Furthermore, thermodynamic characterization of the c-di-GMP → BcsE^217−523^ interaction reveals a binding stoichiometry consistent with two c-di-GMP molecules binding to a single BcsE I-site rather than the apparent inverse stoichiometry observed in the crystals (Fig. 5C versus Fig. 5A; Fig. S3A and B). These results are consistent with both the propensity of c-di-GMP to adopt diverse conformations, including intercalated dimers in solution (2, 3), and the capability of GGDEF I-sites to coordinate both monomeric and dimeric ligands as shown for P. aeruginosa PelD (28, 29) (Fig. S3C and D). Importantly, the dimeric c-di-GMP conformation derived from the solution-based data is also consistent with the reported c-di-GMP conformation necessary for BcsA^PilZ^ domain binding and gating loop displacement during each step of UDP-glucose coordination and cellulose incorporation of the sugar moiety (Fig. S3E and F) (8). We therefore propose that Bcs macrocomplex-bound BcsE could secure the maintenance of a secretion system-proximal pool of c-di-GMP in a BcsA-activating conformation, thus limiting dinucleotide diffusion and boosting processive glucose polymerization.

In line with the monomeric state of BcsE^REC^*^-GGDEF^* in solution, equimolar 2:2:2 BcsE^217−523^RQ heterocomplex assembly appears to be driven by the BcsRQ interactions rather than the BcsE variant itself. To determine how BcsE binds each half of the BcsRQ complex, we designed a series of shorter BcsE variants for copurification assays and identified a GGDEF* domain construct, BcsE^349−523^, covering the interstitial helix α_i_, the GGDEF* domain, and the unstructured C-terminal tail that partakes in stable equimolar interactions with BcsRQ (Fig. 5E and F). We also found that BcsRQ binding is independent of c-di-GMP complexation, as BcsERQ complex reconstitution can be carried out in the absence of dinucleotide and with an I-site mutant incapable of c-di-GMP complexation (R^415^TGD → D^415^TGA). Finally, we show that BcsE^GGDEF^* interacts with BcsQ rather than BcsR, as shown in copurification experiments using individual Bcs^His^R or Bcs^His^Q proteins as baits (Fig. 5F and G). Indeed, a stretch of highly conserved residues distinct from the c-di-GMP binding I-site is found on one side of the BcsE^GGDEF^* module that could have evolved for high-affinity BcsQ binding (Fig. S1B). Importantly, nonoverlapping sites for c-di-GMP and BcsQ complexation would allow both stable assembly of BcsRQ within the Bcs macrocomplex and the possibility of c-di-GMP to migrate in and out of the I-site in a model where the dinucleotide processively switches between the BcsE^GGDEF^* module and BcsA’s PilZ domain for cocatalytic synthase regulation.

### BcsE^NTD^-dependent homooligomerization and binding of conserved Nus antitermination complex components.

**FIG 6:**
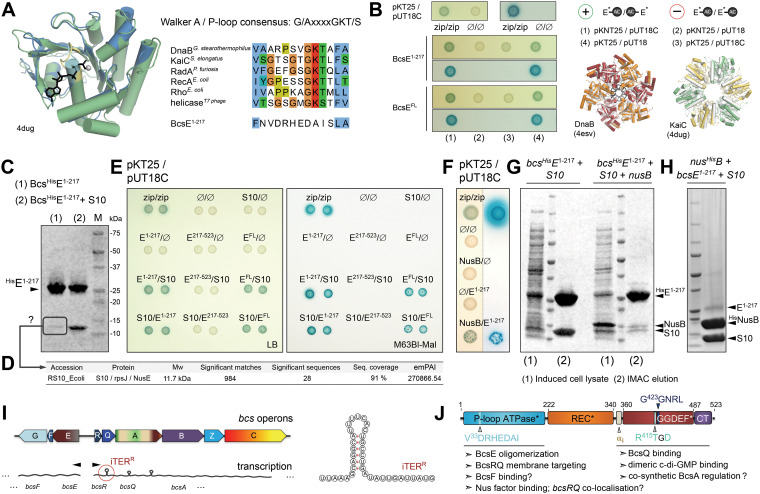
BcsE oligomerization and binding of conserved Nus antitermination complex components. (A) Predicted BcsE^1−217^ RecA-like ATPase fold. (Left) overlay of the prediction model onto Synechococcus elongatus KaiC. (Right) Walker A conservation shown in representative RecA-like ATPases and compared to the corresponding region of BcsE^1−217^. (B) BcsE^1−217^ and BcsE^FL^ homooligomerization. (Left and top right) Bacterial two-hybrid assays using different coexpression strategies for the BcsE-AC fragment fusions. (Bottom right) Overlay of modeled BcsE^1-217^ copies (in gray) with head-to-tail oligomers of RecA-like ATPases in superhelical (DnaB) or ring (KaiC) oligomeric states. (C) IMAC elution fractions of Bcs^His^E^1−217^ when expressed individually or coexpressed with E. coli NusE/S10 protein. (D) Mass spectrometry-based protein identification of a consistently copurifying low-molecular-weight band from an individually expressed, purified, and SDS-PAGE-migrated BcsE^1−217^. (E) Bacterial two-hybrid assay of interactions between BcsE domains and S10/NusE based on plasmid-based adenylate cyclase functional reconstitution in a *cya*-defective E. coli strain (BTH101). The positive zip/zip control is based on coexpressed adenylate cyclase fragments each fused to a homodimerizing leucine zipper region of the yeast protein GCN4. Interactions were evaluated by the growth of blue colonies on X-Gal-supplemented LB (left) or M63BI (right) agar plates. (F) Bacterial two-hybrid assays of BcsE^1−217^-NusB interactions. (G) SDS-PAGE analysis of induced cell lysates, IMAC elution fractions, and S10 copurification upon Bcs^His^E^1−217^ and S10 coexpression in the absence (left) or presence (right) of NusB. (H) IMAC elution fraction upon Nus^His^B, BcsE^1−217^, and S10 coexpression. (I) Organization of the two *bcs* operons and schematic representation of bioinformatically detected potential intrinsic terminators. (Right) Representation of a putative intrinsic terminator in *bcsR*. (J) Results summary showing BcsE domain architecture and proposed functional roles for the identified structural modules.

As mentioned above, the N-terminal region of BcsE (BcsE^1−217^) is predicted to adopt a conserved RecA-like ATPase fold (Fig. 6A). RecA-like motor ATPases are a large family of proteins that use the energy of nucleotide binding and hydrolysis to oligomerize and perform mechanical work in a variety of cellular functions, such as the transport or hydrolysis of proteins (e.g., ABC transporters and proteases) or the binding and remodeling of nucleic acid substrates (e.g., helicases and recombinases) (31).

Structural and sequence alignments of BcsE^NTD^ with catalytically active RecA-like ATPases show severe divergence of key functional motifs (e.g., the ATP/Mg^2+^-coordinating Walker A motif) (Fig. 6A), indicating that BcsE is likely incapable of ATP binding and hydrolysis. Nevertheless, bacterial two-hybrid assays based on split adenylate cyclase (AC) functional reconstitution (32) suggest that BcsE^FL^ is prone to oligomerization and, consistent with the monomeric state of the BcsE^REC^*^-GGDEF^* tandem described above (Fig. 5B), that these interactions are BcsE^NTD^ dependent (Fig. 6B). Interestingly, blue colony growth indicative of BcsE^FL^ and BcsE^NTD^ homooligomerization was only observed in cases when coexpressed AC fragments were fused to different BcsE termini, regardless of their specific type (T25 or T18) (32) or location (N or C terminus) in the fusion constructs. These data suggest that the homotypic BcsE interactions involve different surface regions among the interacting BcsE protomers and are thus consistent with head-to-tail oligomerization mechanisms that are frequently observed in biologically active RecA-like ATPases (Fig. 6B). However, whether BcsE^NTD^ homooligomerization plays a functional role in the above-described targeting of the BcsERQ complex to the inner membrane (Fig. 3) or is involved in additional regulatory processes (see below), remains to be further examined.

Attempts to recombinantly purify the BcsE^1−217^ construct consistently led to the copurification of a second protein species, even in elevated imidazole and salt concentrations in the immobilized-metal affinity chromatography (IMAC) purification buffer (Fig. 6C). Mass spectrometric analyses identified the copurifying species as small ribosomal protein S10, also known as NusE or RpsJ (Fig. 6D). Interestingly, apart from associating with the small ribosomal subunit during protein translation, S10 is also known to moonlight as a key component of the Nus transcription antitermination complex. Nus factors NusA, NusB, S10/NusE, NusG, and SuhB are mostly essential, highly conserved bacterial proteins that are known to associate with and reprogram the transcription apparatus in order to overcome elongation complex dissociation at certain intrinsic and Rho-dependent transcription terminators (33, 34). Although the best-studied examples include N protein-dependent antitermination at early λ phage genes and the regulation of bacterial ribosomal (*rrn*) gene expression (34, 35), a recent study identified conserved NusB-S10 binding sites upstream of additional genes in diverse bacterial species (36). Nevertheless, characterized S10 interactions at the protein-protein level have remained limited to the context of assembled ribosomes or extensively studied transcription antitermination subcomplexes (e.g., see references 35 and 37).

Intrigued by this, we proceeded to assay the putative BcsE-S10 interaction by recombinant coexpression/copurification and cell-based bacterial two-hybrid experiments. We observed that BcsE^NTD^ copurified at an equimolar ratio with overexpressed S10 (pProExHTB-*bcsE^NTD^* plus pRSFDuet1*-*s10* coexpression) and that BcsE likely interacts with S10 *in cellulo* as observed by blue colony growth in the context of both the truncated BcsE^1−217^ construct (BcsE^NTD^) and the full-length BcsE protein (BcsE^FL^) (Fig. 6C and E). We further assayed putative interactions of BcsE^NTD^ with a second Nus factor, NusB, known to interact directly with S10 in the early steps of TAC assembly onto the target mRNA. Whereas bacterial two-hybrid experiments were indicative of weak BcsE^NTD^-NusB interactions *in cellulo*, recombinant coexpression of Bcs^His^E^NTD^ with tag-free S10 and NusB led to the purification of excess Bcs^His^E^NTD^ and only trace amounts of copurifying Nus factors, even if S10 expression levels appeared virtually unchanged (Fig. 6F and G). Conversely, recombinant coexpression of Nus^His^B with tag-free S10 and BcsE^NTD^ led to the purification of an equimolar amount of NusB-S10 complex and trace amounts of a third species, whose molecular weight corresponds to that of BcsE^NTD^ (Fig. 6H). Together, these data indicate that S10 likely uses similar surface regions to interact with its BcsE and NusB partners, whereas higher-affinity NusB complexation could cause competitive remodeling of the equimolar BcsE^NTD^-S10 assemblies and subsequent release of free BcsE.

Although the physiological significance of the observed BcsE-Nus factor interactions remains enigmatic, our findings suggest a possibly broader role for the conserved Nus antitermination machinery than in the well-studied examples of ribosomal or viral gene expression. Interestingly, *in silico* prediction tools (38, 39) detect putative intrinsic terminators within both the *bcsR* and *bcsQ* coding regions. If these potential regulatory elements indeed function as predicted, then the protein-protein interactions observed here could serve to target (via S10 complexation upon exiting the ribosome) and subsequently release (via downstream recruitment of NusB upon antitermination complex assembly) BcsE at the site of *bcsRQ* expression, prior to binding the newly synthesized BcsRQ complex directly and delivering it to the inner membrane for downstream Bcs macrocomplex assembly. Such a hypothesis, however, remains to be experimentally tested.

### Concluding remarks.

Bacteria have evolved complex secretion machineries to deliver large molecules to the cell envelope, external milieu, or host cell targets. Although these systems are typically not essential for bacterial physiology *per se*, they could often provide significant advantages in interspecies competition or be key to a pathogen’s infection cycle. Bacterial exopolysaccharide secretion shares many similarities with the various types of protein secretion systems in that it typically involves intricate signal transduction events to induce the expression and assembly of multiple subunits in order to provide the biosynthetic activities, physical conduit, and energetics for biopolymer extrusion through the complex bacterial envelope (40).

Often, secretion systems are viewed as such at the level of assembled macrocomplexes and substrate extrusion, whereas the initial steps of subunit expression and sequential protein-protein interactions remain largely overlooked. Here, we present the E. coli-like Bcs system as a new candidate paradigm for concerted secretion system assembly and function. We demonstrate that essential-for-secretion BcsR and BcsQ regulate each other’s folding and stability, whereas BcsE packs a subtle but diverse toolkit to fine-tune enterobacterial cellulose production (Fig. 6J). We provide structural and functional data that reveal the protein’s multidomain evolution, fold conservation, and complexation of synthase-activating intercalated c-di-GMP on one hand, together with high-affinity BcsRQ recruitment and facilitated membrane targeting through BcsF interactions on the other. Although more research is needed to uncover physiological roles for the observed BcsE-Nus factor interactions or how the essential BcsRQ subunits control assembly and function of the inner membrane biosynthetic platform, this work lays an important milestone toward more comprehensive models of operon-encoded synthase-dependent polysaccharide secretion in bacterial biofilms.

## MATERIALS AND METHODS

The experiments were not randomized, and the investigators were not blinded during experimental design, execution, or outcome assessment. However, most experiments were reproduced independently by different investigators, including crystallographic, biochemical, biophysical, and phenotypic functional assays.

### Bacterial strains.

Plasmids for recombinant protein expression (see below) were propagated in and isolated from E. coli DH5α cells. All recombinant protein expression for structural and *in vitro* biochemical studies was carried out in BL21(DE3) Star cells, including the expression of selenomethionine-derivatized protein. An E. coli 1094 Δ*bcsQ* strain was used for the complementation phenotypic assays with BcsQ variants expressed from a low-copy-number isopropyl-β-d-thiogalactopyranoside (IPTG)-inducible vector (pAM238; see below). Finally, bacterial two-hybrid experiments were performed using chemically competent BTH101 cells and the IPTG-inducible pKT(N)25 and pUT18(C) expression plasmids with custom-modified multiple cloning sites (see below). All bacterial strains and plasmids used in this study are available upon request.

### Recombinant DNA techniques.

DNA manipulations were carried out using standard protocols for PCR, molecular cloning, transformation, and DNA analyses. Coding regions for BcsR, BcsQ, BcsRQ, BcsE, MinDE, S10, and NusB variants were amplified using E. coli 1094 genomic DNA as a template and a high-fidelity DNA polymerase (Phusion; New England BioLabs) and inserted via digestion/ligation cloning into IPTG-inducible expression vectors with custom-modified multiple-cloning sites (MCS). Point mutations, insertion of stop codons, MCS modifications, and domain deletions within previously reported and newly generated expression constructs were performed using inverse PCR-based protocols and mutation-specific oligonucleotides as primers. All recombinant vectors and introduced mutations were verified by DNA sequencing and, where applicable, IPTG-inducible protein expression.

### Protein expression and purification.

All pProExHTB-encoded constructs (Bcs^His^R, Bcs^His^Q, Bcs^His^RQ, Bcs^His^E^NTD^, Nus^His^B, and Min^His^DE) were expressed as IPTG-inducible variants carrying N-terminal hexahistidine tags cleavable by the human rhinovirus (HRV) 3c protease. BcsQ was also cloned in a standard pET21b vector yielding a C-terminally hexahistidine-tagged protein. As all BcsR (Bcs^His^R) and BcsQ (Bcs^His^Q and BcsQ^His^) constructs failed to yield stable proteins, the coding region corresponding to the BcsRQ tandem was subsequently amplified and cloned into both the pProExHTB and pET21b expression vectors, adding a cleavable N-terminal or noncleavable C-terminal hexahistidine tag to BcsR (pProExHTB-^His^RQ) or BcsQ (pET21b-RQ^His^), respectively. For coexpression studies, the coding region corresponding to full-length tag-free BcsE was cloned into custom-modified pRSFDuet1* expression vector under the control of the first T7 promoter (pRSFDuet1*-BcsE^FL^) (see Table S1 in the supplemental material). Full-length BcsE was also cloned in pProExHTB and pET-^His^SUMO (see below) vectors for standalone expression, but the purified proteins were judged insufficiently stable or pure for structural studies. Based on sequence conservation (PFAM [41]) and predicted tertiary structure (Phyre2, Robetta [22, 23]), several N- and C-terminal deletions of the pRSFDuet1*-BcsE^FL^ construct were tested for expression and copurification with BcsRQ. The coding region for the interacting BcsE^217−523^ construct was subsequently cloned for standalone expression into a modified pET-^His^SUMO plasmid, yielding a hexahistidine-tagged Ulp1-cleavable SUMO moiety fused to the N terminus of the protein of interest (pET-^His^SUMO-BcsE^217−523^). Based on the resulting crystal structure of the BcsE^217−523^ construct, an additional construct corresponding to the C-terminal GGDEF* domain was designed (BcsE^349−523^), and its coding sequence was cloned into the pRSFDuet1* and pET-^His^SUMO expression vectors as described above. The BcsE^1−217^ construct corresponding to the protein’s N-terminal domain was cloned into both pProExHTB and pRSFDuet1* (site 1) vectors. For coexpression of BcsE^1−217^ (in pProExHTB) with S10 and NusB, the coding sequences for the latter were cloned in the first and second sites, respectively, of custom-modified pRSFDuet1* vectors (pProExHTB-Bcs^His^E^1−217^ plus pRSFDuet1*-S10^(site 1)^-NusB^(site 2)^ coexpression; the pRSFDuet1* vector was further modified at the second promoter to introduce unique XhoI and HindIII restriction sites) (Table S1). In addition, a pProExHTB-Nus^His^B plus pRSFDuet1*-BcsE^1-217(site 1)^-S10^(site 2)^ was also employed. For control liposome flotation studies, the coding region for the MinDE tandem was PCR-amplified and cloned into a pProExHTB vector, and MinD was purified as a partner-free protein (Min^His^D) from the clarified cytosolic fraction. Finally, Bcs^His^E^FL^ and Bcs^His^E^FL^F cloned into pProExHTB vectors were used for examining the membrane-targeting role of BcsF. Protein constructs used in the bacterial two-hybrid studies are described separately.

For protein purification, all expression vectors were (co)transformed into chemically competent E. coli BL21(DE3) Star cells. For the expression of native proteins, cells were grown at 37°C under aerobic conditions in terrific broth (TB) medium supplemented with appropriate antibiotics (100 μg/ml ampicillin, 40 μg/ml kanamycin, or a combination of 70 μg/ml ampicillin plus 30 μg/ml kanamycin for coexpressed vectors). At a cell optical density corresponding to an optical density at 600 nm (OD_600_) of 0.8 to 1.0, the cells were moved to 17°C, and overnight protein expression was induced by the addition of IPTG at a final concentration of 0.7 mM. For the expression of selenomethionine-derivatized proteins, 4 liters of cells was initially grown at 37°C in LB medium to an OD_600_ of 0.5 to 0.6. Cells were then pelleted by centrifugation (4,000 × *g*, 15 min, 20°C), gently washed with 200 ml 1× SelenoMet medium base (Molecular Dimensions), collected again, and resuspended in 1 liter complete SelenoMet medium (Molecular Dimensions) supplemented with 40 mg/liter l-selenomethionine and the appropriate antibiotic. Cells were then grown for an additional 1 h at 37°C, transferred to 17°C, and induced with IPTG as described above.

After 16 h, cells were harvested by centrifugation, resuspended in lysis buffer, and flash-frozen in liquid nitrogen. The composition of the lysis buffer was 20 mM HEPES (pH 8.0), 120 mM NaCl, 19 mM imidazole (pH 8.0,) 2 mM β-mercaptoethanol, and 1 tablet/50 ml cOmplete protease inhibitors (Roche) for the Bcs^His^R, Bcs^His^Q, BcsQ^His^, Bcs^His^RQ, Min^His^DE, and Bcs^His^E^217−523^ constructs. For the Bcs^His^RQ-BcsE^FL^, Bcs^His^RQ-BcsE^217−523^, and Bcs^His^RQ-BcsE^349−523^ complexes, the IMAC buffer was also supplemented with 0.5 μM c-di-GMP (Jena Bioscience or Sigma-Aldrich), 2 μM AppCp (Jena Bioscience), 5 mM MgCl_2_, and 10% glycerol. For the expression of Bcs^His^E^1−217^ and the Bcs^His^E^1−217^-S10, Bcs^His^E^1−217^-S10-NusB, and Nus^His^B-S10-BcsE^1−217^ complexes, the concentration of salt in the lysis buffer was increased to 750 mM NaCl.

For all cytosolic protein purifications, cells were thawed and lysed using an Emulsiflex-C3 high-pressure homogenizer (Avestin). Cell debris was removed by centrifugation (1 h at 50,000 × *g* and 4°C), and the cleared lysates were loaded onto buffer-washed Talon Superflow resin (GE Healthcare) at approximately 0.5 to 1 ml of resin per liter of culture. The resin was subsequently washed with more than 20 volumes of IMAC buffer A (protease inhibitor-free lysis buffer as described above), and bound proteins were eluted in a single step with IMAC buffer A supplemented with 200 mM imidazole (pH 8.0) (IMAC buffer B).

For purification of tag-free BcsRQ, eluted protein Bcs^His^RQ protein was supplemented with 15 mM EDTA (pH 8.0) and homemade HRV3c protease at 4°C, concentrated to 2.5 ml using an Amicon Ultra centrifugal filter (30-kDa cutoff; Millipore), desalted using a disposable PD-10 desalting column (GE Healthcare), and incubated overnight for tag removal. The cleaved tag and protease were removed by inverse IMAC, concentrated, and subjected to size exclusion chromatography on a Superdex 200 Increase 10/300 GL column (GE Healthcare) equilibrated in gel filtration buffer (20 mM HEPES [pH 8.0], 120 mM NaCl, and 2 mM dithiothreitol [DTT]). Collected protein fractions were analyzed for purity by SDS-PAGE, pooled, concentrated, flash-frozen in liquid nitrogen, and stored at −80°C.

For purification of tag-free BcsE^217−523^, the eluted ^His^SUMO-fused protein was mixed with homemade yeast protease Ulp1, concentrated to 2.5 ml, desalted on a disposable PD-10 column, and incubated for ^His^SUMO cleavage at 4°C overnight. Cleaved protein was collected in the flowthrough fraction during reverse IMAC on the following day, concentrated, and subjected to size exclusion chromatography on a Superdex 200 Increase 10/300 GL column equilibrated with gel filtration buffer (20 mM HEPES [pH 8.0], 100 mM NaCl, and 2 mM DTT). Collected protein fractions were analyzed for purity, concentrated, aliquoted, and flash frozen for storage at −80°C.

Complexes BcsRQ-BcsE^217−523^ and BcsRQ-BcsE^349−523^ were purified in a similar 2-step IMAC procedure. Eluted proteins were incubated with the viral HRV3c protease for cleavage of the N-terminal hexahistidine tag on BcsR. Imidazole concentrations were lowered via desalting on a disposable PD-10 column, and after overnight incubation at 4°C, the proteins were subjected to size exclusion chromatography using a Superdex 200 Increase 10/300 GL column and gel filtration buffer composed of 20 mM HEPES (pH 8.0), 120 mM NaCl, 5 mM MgCl_2_, 0.5 μM c-di-GMP, 2 μM AppCp, 2 mM DTT, and 10% glycerol. Collected protein fractions were analyzed for purity and stoichiometric complex assembly, concentrated, and flash frozen in liquid nitrogen for storage at −80°C.

To characterize the complex formation and stoichiometry of interaction between BcsRQ and BcsE^FL^, a ternary complex was coexpressed and purified using a similar protocol. However, as the complex appeared to be stabilized by the presence of detergents, after cell lysis, the cell debris was pelleted by slower centrifugation (12,000 × *g*, 15 min, 4°C), and the remaining supernatant was incubated with 0.25% *n*-dodecyl-β-d-maltopyranoside (β-DDM; Anatrace) for 1 h at 4°C. The lysates were then cleared by high-speed centrifugation, and the ternary BcsRQ-BcsE^FL^ complex was purified as the rest of the BcsERQ complexes while keeping a low concentration of detergent (0.06% Cymal-6; Anatrace) in all buffers. For size exclusion chromatography, the Superdex 200 Increase 10/300 GL column was replaced by a Superose 6 Increase 10/300 GL column.

MinD was purified from clarified cytosolic fraction using a single-step metal-affinity purification (IMAC buffer A with 20 mM HEPES [pH 8.0], 120 mM NaCl, and 19 mM imidazole), followed by size exclusion chromatography on a Superdex 200 Increase 10/300 GL column equilibrated with gel filtration buffer (20 mM HEPES [pH 8.0], 100 mM NaCl, and 2 mM DTT). Clean protein fractions were concentrated and flash-frozen for storage at −80°C.

Cells expressing Bcs^His^E^1−217^, Bcs^His^E^1−217^-S10, Bcs^His^E^1−217^-S10-NusB, and Nus^His^B-S10-BcsE^1−217^ were resuspended in high-salt lysis buffer (same as described above but with 750 mM NaCl), and the proteins were purified by a single-step IMAC. The high-salt conditions (750 mM NaCl) were maintained in all buffers.

Finally, expression and purification of the Bcs macrocomplex (pCDFDuet1-Bcs^His^RQA^HA-FLAG^B plus pRSFDuet1*-^Strep^EFG) with various BcsE (Bcs^Strep^E^FL^, Bcs^Strep^E^1−217^, or Bcs^Strep^E^217−523^) and BcsQ (BcsQ or Bcs^HA^Q) variants were performed as reported previously (6). pRSFDuet1*-Bcs^Strep^E^1−217^FG was generated from pRSFDuet1*-Bcs^Strep^E^FL^FG via inverse PCR using two different strategies which yielded consistent results: (i) an insertion of a 4-letter STOP codon following BcsE residue A^217^ (TAAT in DNA) and (ii) a deletion of the REC*-GGDEF* tandem while preserving the ribosome-binding site for *bcsF* to avoid polar effects). pRSFDuet1*-Bcs^Strep^E^217−523^FG was generated by standard restriction/ligase subcloning. Insertion of a hemagglutinin (HA) tag at the N terminus of BcsQ was also conducted by inverse PCR. After expression vector cotransformation, culture growth, and overnight expression induction, cells were pelleted by centrifugation and resuspended in ice-cold lysis buffer containing 20 mM HEPES (pH 8.0), 120 mM NaCl, 10% glycerol, 5 mM MgCl_2_, 10 μM AppCp, 2 μM c-di-GMP, 250 μM cellobiose, 0.5 mg/ml Aspergillus niger cellulase (Sigma-Aldrich), 100 μg/ml lysozyme, and 1 tablet/50 ml cOmplete EDTA-free protease inhibitors (Roche). After lysis (Emulsiflex-C3), cell debris was removed by low-speed centrifugation (12,000 × *g*, 15 min, 4°C), and the membranes were pelleted by ultracentrifugation using an SW 28 Ti Beckman rotor (26,500 rpm, or up to 126,000 × *g*, for 1 h at 4°C). After removal of the supernatant, the membrane fraction was resuspended in solubilization buffer containing all lysis buffer components except lysozyme and cellulase, as well as a mix of detergents at the following final concentrations: 0.4% (wt/vol) digitonin (Sigma-Aldrich), 0.4% (wt/vol) *n*-dodecyl-β-d-maltopyranoside (anagrade β-DDM; Anatrace), 0.4% (wt/vol) decyl maltose neopentyl glycol (DM-NPG; Anatrace), and 0.2% lauryl maltose neopentyl glycol (LM-NPG; Anatrace). After a 60- to 90-min-long incubation at 20°C and under mild agitation, the solubilized membrane fraction was cleared by a second high-speed centrifugation step as described above. The supernatant was then incubated with anti-FLAG M2 affinity gel (50 μl packed resin per liter of induced culture; Sigma-Aldrich) with mild agitation at 4°C for 1 h. After gravity elution of the nonbound fraction, the resin was washed extensively (>30 column bed volumes) with binding buffer containing all lysis buffer components except lysozyme and cellulase, as well as 0.008% (wt/vol) LM-NPG. The bound complexes were then eluted using 4 column bed volumes of elution buffer (affinity buffer supplemented with 3× FLAG peptide at 100 μg/ml) and concentrated on a 100-kDa cutoff Amicon Ultra (MerckMillipore) centrifugal filter.

### SDS-PAGE and Western blot analyses.

Protein fractions were analyzed by standard denaturing SDS-PAGE using 4% to 20% gradient mini-gels (Bio-Rad), Expedeon InstantBlue Coomassie stain, and a Li-Cor Odyssey Fc system for Coomassie visualization (700-nm channel). For Western blot analyses, SDS-PAGE-migrated proteins were directly transferred using a standard mini-gel transfer protocol, 0.2-μm polyvinylidene difluoride (PVDF) membranes, and a Trans-blot Turbo transfer system (Bio-Rad). Blocking and antibody incubations were in the presence of 5% skim milk in Tris-phosphate-buffered saline (TPBS); all washes between and after antibody incubations were with 1× TPBS buffer. Rabbit anti-His_6_ (dilution 1:1,000, ab200537; Abcam) and mouse anti-HA (dilution 1:1,000, number 26183; Thermo Fisher Scientific) antibodies were used as primary antibodies; Alexa Fluor 680-conjugated goat ant-rabbit (dilution 1:10,000, ab175773; Abcam) and donkey anti-mouse (dilution 1:10,000, ab175774; Abcam) were used as secondary antibodies. The Alexa Fluor 680 signal was detected using a Li-Cor Odyssey Fc system in the 700-nm channel.

### Crystallization, data collection, and structure determination.

Crystals were obtained by sitting or hanging-drop vapor diffusion by mixing equal volumes of protein (1.5 to 6 mg/ml) and reservoir solution followed by incubation at 4°C. BcsE^217−523^ crystals also appeared within 3 to 14 days under multiple conditions, with diffracting data sets collected on crystals grown in 100 mM morpholineethanesulfonic acid (MES; pH 6.0), 4% polyethylene glycol 4000 (PEG 4000), 200 mM MgCl_2_, 5% glycerol, and 50 μM c-di-GMP. For cryoprotection, crystals were soaked in reservoir solution supplemented with 25% to 30% glycerol, 1 mM DTT, and 50 μM c-di-GMP. Cryopreserved crystals were flash frozen and stored in liquid nitrogen. Data were collected on frozen crystals at 100 K using synchrotron radiation at beamlines PX1 and PX2 at the Soleil synchrotron.

Data reduction was carried out with the software package XDS (42). Experimental phases were obtained by single-wavelength anomalous diffraction (SAD) experiments on crystals grown from selenomethionine-derivatized protein and with wavelengths corresponding to the experimentally determined selenium K-edge. Initial BcsE^217−523^ models were obtained using the automated model building tools of PHENIX and Buccaneer (43, 44). Reiterative refinements in PHENIX, COOT, and BUSTER yielded the final refined model (43, 45, 46). Data collection and refinement statistics are summarized in Table S2. For illustration purposes, all crystal structures were displayed with the PyMol Molecular Graphics System (Schrödinger, LLC) or UCSF Chimera (47). The latter was also used for displaying the 3D reconstructions of the assembled Bcs macrocomplex.

### Single-particle electron microscopy.

Negative-stain single-particle electron microscopy was used for visualization of various Bcs proteins and protein complexes. Briefly, 5 μl of eluted samples (concentration, ∼0.01 to 0.05 mg/ml) were spotted on glow-discharged carbon-coated copper grids (Agar Scientific). After a 1-min incubation, the extra liquid was blotted off, and the grids were passed sequentially through three drops of 2% (wt/vol) uranyl acetate solution, with a second incubation in the last drop before blotting and air drying. Micrographs were taken on a Thermo Fisher Scientific T12 Tecnai electron microscope operated at 100 kV accelerating voltage and equipped with a LaB6 filament and a K2 Base direct electron detector. For the protein complexes purified from cells expressing Bcs^His^RQA^HA-FLAG^B plus Bcs^Strep^E^FL^FG or Bcs^His^RQA^HA-FLAG^B plus Bcs^Strep^E^217−523^FG, particles were autopicked in EMAN2 (48), saved as .box coordinates, and converted into a .star particle stack in Relion2 (49). Micrograph contrast transfer function (CTF) correction and two-dimensional (2D) classification were performed in cryoSPARC v2 after particle reextraction using the Relion2-generated .star file as metadata input (50). A total of 3,810 particles were classified for the Bcs macrocomplex carrying full-length BcsE (control) and 6,242 particles for the complex purified from cells expressing the BcsE^217−523^ truncated variant.

### Protein identification by mass spectrometry.

Coomassie-stained gel bands were excised and subjected to in-gel enzymatic digestion. Briefly, the bands were extensively washed with acetonitrile and 100 mM NH_4_HCO_3_, dried, and treated with 10 mM DTT at 56°C for 30 min. After DTT removal, cysteine carbamidomethylation was performed at room temperature for 30 min by the addition of 55 mM iodoacetamide. The washing procedure was then repeated, the gel slices were dried, and the proteins were digested overnight at room temperature by the addition of 20 μl/band of 10 ng/μl Porcine Gold trypsin (Promega) diluted in 50 mM NH_3_HCO_3_. Peptides were extracted in two steps: first, with 20 μl of 50% acetonitrile-0.1% formic acid solution and, second, with 20 μl of 100% acetonitrile. Peptides were vacuum dried and resuspended in 5% acetonitrile-0.1% trifluoroacetic acid (TFA) prior to nanoscale liquid chromatography-tandem mass spectrometry (nanoLC-MS/MS) analyses. The latter were performed with a TripleTOF 4600 mass spectrometer (Sciex) coupled to an UltiMate 3000 RSLCnano system (Thermo Fisher Scientific). Peptides were first desalted on an Acclaim Pepmap 100 C_18_ reverse-phase precolumn (3 μm, 100 Å, 75-μm inside diameter [i.d.], 2-cm length) using a loading buffer containing 2% acetonitrile and 0.05% TFA in water and a flow rate of 5 μl/min. A second Acclaim Pepmap 100 C_18_ column (2 μm, 100 Å, 75-μm i.d., 50-cm length) was then used as an analytical column, and bound peptides were eluted from the reverse phase using a 5% to 35% solvent B gradient for 40 min at a flow rate of 300 nl/min (solvent A, 0.1% formic acid in water; solvent B, 0.1% formic acid in 100% acetonitrile). nanoLC-MS/MS experiments were conducted using data-dependent acquisition by selecting the 20 most intense precursors for collision-induced dissociation (CID) fragmentation with the Q1 quadrupole set to low resolution for increased sensitivity. Raw data were processed using proprietary MS data converter software (Sciex), and protein identification was performed using the Mascot search engine (Matrix Science) against the E. coli taxon in the Swiss-Prot database and with carbamidomethylation of cysteines set as fixed modification. Oxidation of methionines was set as variable modifications. Peptide and fragment tolerances were set at 25 ppm and 0.05 Da, respectively. Only peptides with a Mascot score higher than the identity threshold (30) at less than 1% of false-positive discovery rate were considered.

### Size exclusion chromatography coupled with static multiangle light scattering.

For size exclusion chromatography coupled with static multiangle light scattering (SEC-MALS), purified proteins or protein complexes were subjected to gel filtration using a Superose 6 Increase 10/300 GL column (GE Healthcare) for the BcsRQ-BcsE^FL^ complex or a Superdex 200 Increase 10/300 GL column for all other samples. The columns were preequilibrated with the respective gel filtration buffers. For the BcsE^217−523^ construct, the protein was analyzed in its apo form, as well as following incubation with 2-fold excess c-di-GMP. The high-performance liquid chromatography (HPLC) system (Shimadzu) was coupled to a 3-angle light-scattering detector (miniDAWN TREOS) and a refractive index detector (Optilab rEX) (Wyatt technology). For each experiment, the system was preequilibrated overnight with gel filtration buffer and at the desired flow rate. Data were collected every second at a flow rate of 0.5 ml/min and were analyzed using the ASTRA software to obtain the molar mass and polydispersity of the sample across the protein elution peaks. For detector normalization and data quality control, purified delipidated bovine serum albumin (BSA; Sigma) was used prior to each experiment.

### Protein complex reconstitution using purified proteins.

Purified BcsRQ complex was incubated with excess BcsE^217−523^-D^415^TGA, and after a 15-min incubation on ice, the proteins were subjected to size exclusion chromatography using a Superdex 200 Increase 10/300 GL column and using c-di-GMP-free gel filtration buffer (20 mM HEPES [pH 8.0], 100 mM NaCl, and 2 mM DTT). The purified BcsRQ and BcsE^217−523^-D^415^TGA proteins were also injected on their own in separate chromatography runs. BcsRQ-BcsE^217−523^-D^415^TGA complex formation was detected by depletion of the BcsRQ peak and appearance of a new *A*_280_ peak shifted toward the front of the column.

### Isothermal titration calorimetry.

Apparent dissociation constants (*K_d_*) and stoichiometry of interactions (N) were measured by isothermal titration calorimetry (ITC) using a Microcal VP-ITC calorimeter from Malvern Panalytical at 20°C. For c-di-GMP binding studies, 0.8 to 1 mM c-di-GMP was used as a ligand in the syringe, and 50 μM purified protein was added to the cuvette. The proteins and ligand were purified/diluted in the exact same buffer to minimize nonspecific dilution heat effects. Protein concentrations were determined by a combination of methods, including a reducing agent-compatible colorimetric assay (RC DC; Bio-Rad) and 280-nm absorbance measurements under denaturing conditions (*A*_280_; 6 M guanidinium chloride), while accounting for potential scattering contributions (*A*_330_). For BcsRQ-BcsE^217−523^ complex formation, tag-free BcsRQ and BcsE^217−523^ were purified in the same buffer (20 mM HEPES [pH 8.0], 100 mM NaCl, and 2 mM DTT), and 10-fold more concentrated BcsRQ (180 μM) was titrated from the syringe into BcsE^217−523^ (18 μM) in the cuvette. All ITC data were analyzed by integrating the injection heat effects, normalized to the amount of ligand and protein present, and curve fitting based on a single-site binding model using the Origin software package for Microcal. For all titrations, titrations of the ligand into buffer were performed to account for heat dilution effects, and the latter were subtracted during the ligand binding analysis. The apparent dissociation constants (*K_d_*) and stoichiometries of interaction (N) were derived from the data by using standard procedures, and the graphs were replotted using GraphPad Prism software.

### Calcofluor-binding cellulose secretion assay.

To test for the functional effects of the C-terminal BcsQ deletion (BcsQ^ΔC10^), chemically competent cells were prepared from an E. coli 1094 Δ*bcsQ* deletion strain (6). The latter was transformed with a low-copy-number plasmid (pAM-238) carrying wild-type or mutant *bcsQ* genes and plated on LB agar plates (Miller) supplemented with the appropriate antibiotics (60 μg/ml streptomycin and 15 μg/ml chloramphenicol). Single colonies were inoculated in 5 ml LB medium with antibiotics and left to grow overnight at 37°C with agitation. On the following morning, 5 μl of each culture was spotted onto low-salt LB agar plates (1.5 g/liter NaCl) supplemented with the antibiotics, 0.1 mM IPTG, and 0.02% calcofluor (fluorescent brightener 28; Sigma-Aldrich). The spots were allowed to air dry, and the plates were incubated at 30°C. After 24 h, the plates were photographed under brief illumination with long-wave UV light (365 nm).

### Bacterial two-hybrid assay.

We used the adenylate cyclase two-hybrid complementation assay to probe protein-protein interactions (32). We first custom modified the standard expression vectors to introduce BamHI and KpnI cloning sites in the respective MCS while optimizing the number and type of exogenous amino acids to be added to the recombinant hybrids by PCR amplification and restriction digestion of the products (Table S1). An intrinsic KpnI site in the BcsE^FL^ coding region was also modified by introducing a silent mutation in the pRSF-Duet-BcsE^FL^ construct through inverse PCR. Coding regions for BcsE, S10, and NusB full-length proteins or truncated variants were then PCR amplified with primers carrying the corresponding restriction sites, digested, and ligated into the modified vectors. All recombinant constructs were amplified in DH5α cells and verified by DNA sequencing.

The bacterial two-hybrid assay was performed using standard protocols (32). Briefly, chemically competent E. coli BTH101 cells were cotransformed with derivatives of the pUT18(C) and pK(N)T25 vectors and plated on LB Miller agar supplemented with 100 μg/ml ampicillin and 40 μg/ml kanamycin. Individual cotransformant colonies were picked and grown overnight at 37°C in liquid antibiotic-supplemented LB medium. The next morning, 4 μl of saturated culture was spotted onto LB Miller agar (supplemented with 100 μg/ml ampicillin, 40 μg/ml kanamycin, 0.1 mM IPTG, and 40 μg/ml 5-bromo-4-chloro-3-indolyl-β-d-galactopyranoside [X-Gal]) or M63BI agar (supplemented with 50 μg/ml ampicillin, 25 μg/ml kanamycin, 0.1 mM IPTG, 40 μg/ml X-Gal, and 0.2% maltose) plates. Protein interactions were evaluated after approximately 30 h of incubation at 30°C by blue colony color in the case of LB Miller agar plates and by both colony growth and blue color in the case of M63BI agar plates. pUT18(C) and pK(N)T25 vectors carrying only the AC fragment coding sequences were used in cotransformations as negative controls, whereas cotransformants expressing pKT25-zip and pUT18C-zip vectors were used as positive controls. The latter vectors are derivatives of the pK(N)T25 and pUT18(C) vectors in which the leucine zipper of Gcn4 is genetically fused in frame to the T25 and T18 adenylate cyclase fragments, respectively. The results are representative of at least 3 independent experiments and 6 biological replicates.

### Liposome flotation experiments.

We used liposome flotation to monitor the correlation between the distributions of fluorescently labeled liposomes and Coomassie-stained proteins across sucrose density gradients. For BcsRQ membrane binding studies (Fig. 3B), we used extruded liposomes prepared from commercially available E. coli total membrane lipids (Avanti Polar Lipids, Inc.) at stock concentration of 10 mg/ml in buffer containing 120 mM NaCl and 20 mM HEPES (pH 8.0). For lipid detection, 1% (vol/vol) of egg l-α-phosphatidylethanolamine-*N*-(7-nitro-2-1,3-benzoxadiazol-4-yl) (NBD-PE; Avanti Polar Lipids, Inc.) was added to the mix prior to liposome preparation. For this, powdered lipids were hydrated with buffer and subjected to multiple cycles of flash freezing in liquid nitrogen, thawing at 50°C, sonication, and 0.1-μm filter extrusion. Liposomes and purified protein (BcsRQ or, as a positive control, MinD) were mixed in 100 μl of the same buffer at final concentration of 1 mg/ml each, and this volume was then premixed with the heavy fraction of a sucrose density gradient (3 ml of 80% sucrose in buffer) in 13.2-ml ultracentrifuge tubes. The heavy fraction was then layered with 6 ml 60% sucrose in buffer, which in turn was layered with 3 ml 10% sucrose in buffer without mixing the gradient layers. The samples were then subjected to ultracentrifugation in a Beckman Coulter SW 41 Ti rotor for 16 h at 35,000 rpm and 4°C. One-milliliter aliquots were then gently removed from the top, and the NBD fluorescence for each fraction was measured with excitation at 465 nm and emission detection at 535 nm using a Perkin Elmer LS-50B luminescence spectrometer. In parallel, protein distribution along the gradient aliquots was visualized by denaturing SDS-PAGE. Additions of ADP-Mg^2+^, ATP-Mg^2+^, AppCp-Mg^2+^, and cardiolipin were tested but not found to affect BcsRQ distribution along the density gradients. Results are representative of at least 3 experiments with 2 technical replicates each.

For Bcs^His^E^FL^ versus Bcs^His^E^FL^F localization (Fig. 3K), E. coli Bl21(DE3) Star cells overexpressing the respective constructs from pProExHTB expression vectors were lysed using an Emulsiflex-C3 homogenizer in buffer containing 20 mM HEPES (pH 8.0), 120 mM NaCl, 10% glycerol, 1 μM c-di-GMP, and 1 tablet/50 ml cOmplete Protease inhibitor cocktail. Cell debris was removed by centrifugation at 12,000 × *g* and 4°C for 10 min, and the membrane fraction was then pelleted from the supernatant by ultracentrifugation at 35,000 rpm for 75 min at 4°C using a Beckman Coulter SW 41 Ti rotor. After removing the supernatant, the membranes were resuspended with a Potter-Elvehjem homogenizer in the same buffer, and after a second ultracentrifugation step, the pelleted membranes were weighed and resuspended at 20 mg/ml final concentration, and 1% (vol/vol) egg NBD-PE was added to trace the lipid distribution. The total membrane protein content was analyzed by SDS-PAGE and Western blotting, leading to identification of the Bcs^His^E^FL^ protein band enriched in the membrane fractions of BcsF-coexpressing cells (pProExHTB-^His^E^FL^F coexpression). These membrane fractions were subjected to several cycles of low-intensity sonication for liposome generation, and 100 μl of each replicate was mixed with the heavy fraction of a sucrose density gradient (3 ml 80% sucrose in buffer) and subjected to gradient ultracentrifugation as described above. Native proteoliposome distribution was visualized via detection of NBD fluorescence along the gradient aliquots, whereas Bcs^His^E^FL^ distribution was visualized by SDS-PAGE and Coomassie staining (Fig. 3L). Results for all flotation experiments are representative of at least 3 independent experiments with 2 technical replicates each.

### Protein and RNA structure prediction tools.

Protein conserved domain detection and tertiary structure prediction were carried out using the NCBI BLASTP suite (https://blast.ncbi.nlm.nih.gov/Blast.cgi), PFAM database (https://pfam.xfam.org), Phyre2 (http://www.sbg.bio.ic.ac.uk), and Robetta servers (http://new.robetta.org) (22, 23, 41, 51). A search for structural homologues in the Protein Data Bank was carried out using the DALI server (http://ekhidna2.biocenter.helsinki.fi/dali/) (25). Potential intrinsic terminators in mRNA were predicted using the ARNold (http://rssf.i2bc.paris-saclay.fr/toolbox/arnold/) and iTERM-PseKNC (http://lin-group.cn/server/iTerm-PseKNC/) tools (38, 39).

### Data availability.

Crystallographic structure factors and coordinates have been deposited in the RCSB Protein Data Bank (https://www.rcsb.org/) with accession code 6TJ0.
