# Supplementary material for: Structure and Multitasking of the c-di-GMP-Sensing Cellulose Secretion Regulator BcsE
Source: mBio. 2020 Aug 11;11(4):e01303-20. doi: 10.1128/mBio.01303-20 (PMC7439463; doi:10.1128/mBio.01303-20)
Supplement: TABLE S1 [file mBio.01303-20-st001.pdf]

Expression constructs	Proteins	Primers	Ref.
1. pProExHTB-$bcs^{His}R$	Bcs^{His}R: MSYYHHHHHHDYDIPTTLEVLFQ-✂-GPMGSM^{1}… ; 10.4 kDa (with tag)	s: CATGTAGGATCCATGAATAACAATGAACCAGATACTCTGCCTGATCCC (BamHI)<br>as: CTAGATGCGGCCGCCTACTTTTGTTGCGCAAACTCTGCCAG (NotI)	This study
2. pProExHTB-$bcs^{His}Q$	Bcs^{His}Q: MSYYHHHHHHDYDIPTTLEVLFQ-✂-GPMGSM^{1}… ; 31.3 kDa (with tag)	s: CATATGGGATCCATGGCCGTACTGGGATTGCAG (BamHI)<br>as: CTTGATGCGGCCGCTCATGATTTACTCCCGACTGGCGTTTTCAGC (NotI)	This study
3. pProExHTB-$bcs^{His}RQ$	Bcs^{His}R: MSYYHHHHHHDYDIPTTLEVLFQ-✂-GPMGSM^{1}… ; 10.4 kDa (with tag)<br>BcsQ: wild-type, full-length, tag-free protein ; 27.9 kDa	s: CATGTAGGATCCATGAATAACAATGAACCAGATACTCTGCCTGATCCC (BamHI)<br>as: CTTGATGCGGCCGCTCATGATTTACTCCCGACTGGCGTTTTCAGC (NotI)	This study
4. pET21b-$bcsQ^{His}$	BcsQ^{His}: M^{1} … TPVGSKS^{250}AAALEHHHHHH ; 29.2 kDa (with tag)	s: GTATACCATATGGCCGTACTGGGATTGCAGGG (NdeI)<br>as: CTTGATGCG GCC GCTGATTTACTCCCGACTGGCGTTTTCAGC (NotI)	This study
5. pET21b-$bcsRQ^{His}$	BcsR: wild-type, full-length protein, tag-free protein ; 7 kDa<br>BcsQ^{His}: M^{1} … TPVGSKS^{250}AAALEHHHHHH ; 29.2 kDa (with tag)	s: GGATCCCATATGAATAACAATGAACCAGATACTCTGCC (NdeI)<br>as: CTTGATGCG GCC GCTGATTTACTCCCGACTGGCGTTTTCAGC (NotI)	This study
6. pET21b-$bcsRQ^{C39AD41A\text{-}His}$	same as BcsRQ^{His} ;<br>point mutations C39AD41A in BcsQ: … V^{36}DAAPANLLRLSFN …	s: GGCCAACTTGTTGCGCCTGTCATTTAACGTTGATTTTAC<br>as: GGGGCGGCATCGACCACCAGGACATTTTCTCC (inverse PCR on pET21b-$bcsRQ^{His}$)	This study
7. pET21b-$bcsRQ^{C39AD41AL43D\text{-}His}$	same as BcsRQ^{His} ;<br>point mutations C39AD41AL43D in BcsQ: … V^{36}DAAPANDLRLSFN …	s: CCAACGACTTGCGCCTGTCATTTAACGTTGATTTTACCC<br>as: CCGGGGCGGCATCGACCACCAGGACATTTTCTCCCAACATTTG (inverse PCR as in 6.)	This study
8. pAM238-$bcsQ$	BcsQ: M^{1}…S^{250} ; 27.9 kDa	pAM238 vector amplification:<br>s: GATTGGTGAAGCTTACTGGCCGTCGTTTTACAACG (HindIII)<br>as: CCCTATGAATTCGGCGTAATCATGGTCATAGCTG (EcoRI)<br>*bcsQ* cloning (with own ribosome-binding site):<br>s: CATATGGAATTCAAGTAGGGGATTGGTGAATGGCCGTACTGGGATTGCAG (EcoRI)<br>as: CTCGAGAAGCTTTCATGATTTACTCCCGACTGGCGTTTTCAGC (HindIII)	This study
9. pAM238-$bcsQ^{\Delta C10}$	Bcs$^{\Delta C10}$Q: M^{1}…S^{240} ; 27 kDa	Strategy 1. STOP codon insertion after S^{240} (inverse PCR on 8.):<br>s: GGCTGAAAACGCCAGTCGGGAGTAAATCATGA<br>as: CTCAGGAATAGTTCAACAGGCACCAGTTCGCCAG<br>Strategy 2. STOP codon insertion and $bcsQ^{C10}$ deletion (inverse PCR on 8.)<br>as: GGAATAGTTCAACAGGCACCAGTTCGCC<br>s: TGAAGCTTACTGGCCGTCGTTTTACAACGTC	This study
10. pProExHTB-$bcs^{His}E^{FL}$	Bcs^{His}E^{FL}: MSYYHHHHHHDYDIPTTLEVLFQ-✂-GPMGSM^{1}… ; 62.8 kDa (with tag)	s: CATATGGGATCCATGAGGGACATTGTGGACCCTGTATTC (BamHI)<br>as: CTCGAGGCGGCCGCTCATGATGAGCGCTCCACAGCATC (NotI)	This study
11. pProExHTB-$bcs^{His}E^{FL}F$	Bcs^{His}E^{FL}: same as in 10.<br>BcsF: wild-type, full-length, untagged protein ; 7.4 kDa	s: CATATGGGATCCATGAGGGACATTGTGGACCCTGTATTC (BamHI)<br>as: CTCGAGGCGGCCGCTCATTTTTTGGTTGCCCTGGCTTTTTCCGTG (NotI)	This study
12. pET-^{His}SUMO-$bcsE^{FL}$	MGSSHHHHHH-SUMOtag-GG-✂-SM^{1}… ; 73 kDa (with tag), 59.5 kDa after cleavage	s: CATATGGGATCCATGAGGGACATTGTGGACCCTGTATTC (BamHI)<br>as: CTCGAGGCGGCCGCTCATGATGAGCGCTCCACAGCATC (NotI)	This study
13. pRSFDuet1*-$bcsE^{FL}$	MGSM^{1} … ; full-length, untagged protein, 59.7 kDa	Vector MCS1 redesign (His-tag removal, redesign of BamHI/NotI sites)::<br>s: CTCGAGGCGGCCGCATAATGCTTAAGTCGAACAGA (NotI)<br>as: CATATGGGATCCCATGGTATATCTCCTTATTAAAG (BamHI)<br>$bcsE^{FL}$ cloning:<br>s: CATATGGGATCCATGAGGGACATTGTGGACCCTGTATTC (BamHI)<br>as: CTCGAGGCGGCCGCTCATGATGAGCGCTCCACAGCATC (NotI)	This study
14. pRSFDuet1*-$bcsE^{1\text{-}217}$	MGSM^{1}… A^{217} ; untagged BcsE^{NTD}, 25.1 kDa	Vector MCS1 redesign: as in 13. $bcsE^{1\text{-}217}$ cloning:<br>s: CATATGGGATCCATGAGGGACATTGTGGACCCTGTATTC (BamHI)<br>as: CTCGAGGCGGCCGCCTACGCCTCTTCGCTTTGAACTAATGTC (NotI)	This study
15. pRSFDuet1*-$bcsE^{217\text{-}523}$	MGSA^{217}… ; untagged BcsE$^{REC*\text{-}GGDEF*}$, 35 kDa	Vector MCS1 redesign: as in 13. $bcsE^{217\text{-}523}$ cloning:<br>s: ACCATGGGATCCGCGGAGATCCAACCACGCAGC (BamHI)<br>as: CTCGAGGCGGCCGCCTACGCCTCTTCGCTTTGAACTAATGTC (NotI)	This study
16. pRSFDuet1*-$bcsE^{217\text{-}523}$-D^{415}TGA	same as 14. ; I-site targeted mutagenesis R^{415}TGD→D^{415}TGA	s: GGCGCTATCATGACCATTGGCGGTAATC<br>as: GGTATCGTTAGGGCGACACAGCGTCAG (inverse PCR)	This study
17. pRSFDuet1*-$bcsE^{217\text{-}340}$	MGSA^{217}… G^{340} ; untagged, 14.1 kDa	Vector MCS1 redesign: as in 13. $bcsE^{217\text{-}340}$ cloning:<br>s: ACCATGGGATCCGCGGAGATCCAACCACGCAGC (BamHI)<br>as: CTCGAGGCGGCCGCCTACCCTTGCACGCTTTCGATCATCGTC (NotI)	This study
18. pRSFDuet1*-$bcsE^{217\text{-}347}$	MGSA^{217}… V^{347}; untagged, 15 kDa	Vector MCS1 redesign: as in 13. $bcsE^{217\text{-}347}$ cloning:<br>s: ACCATGGGATCCGCGGAGATCCAACCACGCAGC (BamHI)<br>as: CTCGAGGCGGCCGCCTACACATAGCGACTAAACTTCTGCCCTTGC (NotI)	This study
19. pRSFDuet1*-$bcsE^{217\text{-}359}$	MGSA^{217}… V^{359}; untagged, 16.3 kDa	Vector MCS1 redesign: as in 13. $bcsE^{217\text{-}359}$ cloning:<br>s: ACCATGGGATCCGCGGAGATCCAACCACGCAGC (BamHI)<br>as: CTCGAGGCGGCCGCCTACTGGGTCATTGACAGCAAGGTAGTGATATC (NotI)	This study
20. pRSFDuet1*-$bcsE^{349\text{-}523}$	MGSE^{349}… ; untagged BcsE^{GGDEF*}, 20.1 kDa	Vector MCS1 redesign: as in 13. $bcsE^{349\text{-}523}$ cloning:<br>s: ACCATGGGATCCGAAGATATCACTACCTTGCTGTCAATGACCCAG (BamHI)<br>as: CTCGAGGCGGCCGCTCATGATGAGCGCTCCACAGCATC (NotI)	This study
21. pRSFDuet1*-$bcsE^{359\text{-}523}$	MGSQ^{359}…; untagged, 19 kDa	Vector MCS1 redesign: as in 13. $bcsE^{359\text{-}523}$ cloning:<br>s: ACCATGGGATCCCAGCCGCTCAAACTGCGTGGTTTC (BamHI)<br>as: CTCGAGGCGGCCGCTCATGATGAGCGCTCCACAGCATC (NotI)	This study

22. pET-^{His}SUMO-*bcsE*$^{217-523}$	MGSSHHHHHH-SUMOtag-GG-✂-SA^{217}… ; 48 kDa (with tag), 34.7 after cleavage	s: ACCATGGGATCCGCGGAGATCCAACCACGCAGC (BamHI)<br>as: CTCGAGGCGGCCGCCTACGCCTCTTCGCTTTGAACTAATGTC (NotI)	This study
23. pET-^{His}SUMO-*bcsE*$^{217-523}$-*D*^{415}*TGA*	same as 21. ; I-site targeted mutagenesis R^{415}TGD→D^{415}TGA	same as in 16.	This study
24. pCDFDuet1-*bcs*^{His}*RQA*$^{HA-FLAG}$*B*	Bcs^{His}R: MGSSHHHHHHHHAAGSN^{2}NNE… ; 8.6 kDa (with tag)<br>BcsQ: wild-type, full-length, untagged BcsQ ; 27.9 kDa<br>BcsA$^{HA-FLAG}$: … Q^{872}GSARSSGRTGLEFEEFYPYDVPDYAADYKDDDDKRS ; 103.9 kDa (with tags)<br>BcsB: wild-type, full-length, untagged BcsB ; 86.1 kDa	Vector MCS1 redesign for PstI/NotI restriction/ligation cloning<br>s: GCGCAACAAAAGTAGGCGGCCGCATAATGCTTAAGTCGAACAGA (NotI)<br>as: TCGATCCTGCAGCGTGATGGTGGTGATGATGGTGATGGCTGCTGCCCATG (PstI)<br>Template DNA: *E. coli* 1094 *bcsA*$^{HA-FLAG}$ genomic DNA<br>*bcs*^{His}*RQA*$^{HA-FLAG}$*B* cloning:<br>s: CATCACGCTGCAGGATCGAATAACAATGAACCAGATACTC (PstI)<br>as: CGACTTAAGCATTATGCGGCCGCTTACTCGTTATCCGGGTTAAGACG (NotI)	6
25. pCDFDuet1-*bcs*^{His}*R*^{HA}*QA*$^{HA-FLAG}$*B*	Bcs^{His}R, BcsA$^{HA-FLAG}$, BcsB: as in 24.<br>Bcs^{HA}Q: M^{1}YPYDVPDYAGSGAGSGTGA^{2}VL… ; 29.6 kDa (with tag)	s: GTAGCGGTGCCGGTAGCGGTACAGGTGCCGTACTGGGATTGCAGG<br>as: CTGCATAATCCGGAACATCATACGGATACATTCACCAATCCCCTACTTTTGTTGC<br>(inverse PCR on 24.)	This study
26. pRSFDuet1*-^{STREP}*E*^{FL}*FG*	Bcs^{STREP}E^{FL}: MASWSHPQFEKGSM^{1}…<br>BcsF: wild-type, full-length, untagged protein ; 7.4 kDa<br>BcsG: wild-type, full-length, untagged protein ; 59.6 kDa	*bcsE*^{FL}*FG* cloning in pRSFDuet1* (vector redesign as in 13.)<br>s: CATATGGGATCCATGAGGGACATTGTGGACCCTGTATTC (BamHI)<br>as: CTCGAGGCGGCCGCCTACTGCATTTGAGTTCTCGGAGAC (NotI)<br>STREP II tag insertion (inverse PCR):<br>s: CCGCAGTTCGAAAAAGGATCCATGAGGGACATTGTGGACCCTGTATTC<br>as: GTGGCTCCAGCTAGCCATGGTATATCTCCTTATTAAAG	6
27. pRSFDuet1*-^{STREP}*E*$^{217-523}$*FG*	Bcs^{STREP}E$^{217-523}$: MASWSHPQFEKGSA^{217}EIQPRS… ; 36.1 kDa	Insert replacement in 26.<br>s: ACCATGGGATCCGCGGAGATCCAACCACGCAGC (BamHI)<br>as: CTCGAGGCGGCCGCCTACTGCATTTGAGTTCTCGGAGAC (NotI)	This study
28. pRSFDuet1*-^{STREP}*E*$^{1-217}$*FG*	Bcs^{STREP}E$^{1-217}$: MASWSHPQFEKGSM^{1}…A^{217} ; 26.3 kDa	Strategy 1. 4-letter STOP codon insertion after A^{217} (inverse PCR on 26.):<br>s: ATGCGGAGATCCAACCACGCAGCGACG<br>as: TAAGCCTCTTCGCTTTGAACTAATGTCCAGATACCATTTTGTTGC<br>Strategy 2. STOP codon insertion and *BcsE*$^{REC*-GGDEF*}$ deletion (inverse PCR on 26.):<br>s: ATGAATACCAGAACCCATGCGACTGTTAGATGATGCTG<br>as: TAAGCCTCTTCGCTTTGAACTAATGTCCAGATACCATTTTGTTGC	This study
29. pKT25-*zip*	T25-GCN4^{ZIP} (…IQRMKQLEDKVEELLSKNYHLENEVARLKKLVGER…)	Vector control used as commercially available (Euromedex)	32
30. pKT18C-*zip*	T18-GCN4^{ZIP} (…IQRMKQLEDKVEELLSKNYHLENEVARLKKLVGER…)	Vector control used as commercially available (Euromedex)	32
31. pKT25-*bcsE*$^{1-217}$	T25-GSTLEGS-M^{1}… A^{217} ; 49.9 kDa	Vector MCS redesign:<br>s: ACTATGGGTACCCTAAGAATTCGGCCGTCGTTTTACAACGTCG (KpnI)<br>as: CATATGGGATCCCTCTAGAGTCGACCCTGCAGCC (BamHI)<br>*bcsE*$^{1-217}$ cloning:<br>s: CATATGGGATCCATGAGGGACATTGTGGACCCTGTATTC (BamHI)<br>as: ACTATGGGTACCATTACGCCTCTTCGCTTTGAACTAATGTCCAGATACC (KpnI)	This study
32. pKNT25-*bcsE*$^{1-217}$	MGSM^{1}… A^{217}-GTSNSMT-T25 ; 50.3 kDa	Vector MCS redesign:<br>s: ACTATGGGTACCTCGAATTCAATGACCATGCAGCAATCGCATC (KpnI)<br>as: CATATGGGATCCCATAGCTGTTTCCTGTGTGAAATTGTTATCC (BamHI)<br>*bcsE*$^{1-217}$ *cloning:*<br>s: CATATGGGATCCATGAGGGACATTGTGGACCCTGTATTC (BamHI)<br>as: ACTATGGGTACCCGCCTCTTCGCTTTGAACTAATGTCCAGATACC (KpnI)	This study
33. pUT18-*bcsE*$^{1-217}$	MGSM^{1}… A^{217}-GTSNS-T18 ; 44.9 kDa	Vector MCS redesign:<br>s: ACTATGGGTACCTCGAATTCAGCCGCCAGCGAGG (KpnI)<br>as: CATATGGGATCCCATAGCTGTTTCCTGTGTGAAATTGTTATCC (BamHI)<br>*bcsE*$^{1-217}$ *cloning:* same as 32.	This study
34. pUT18C-*bcsE*$^{1-217}$	T18-HCRSTGS-M^{1}… ; 45.4 kDa	Vector MCS redesign:<br>s: ACTATGGGTACCGTGCACTCTCAGTACAATCTGCTCTGATGC (KpnI)<br>as: CATATGGGATCCAGTCGACCTGCAGTGGCGTTCC (BamHI)<br>*bcsE*$^{1-217}$ cloning: same as 31.	This study
35. pKT25-*bcsE*^{FL}	T25-GSTLEGS-M^{1}… ; 84.4 kDa	Vector MCS redesign: as in 31. *bcsE*^{FL} cloning:<br>s: CATATGGGATCCATGAGGGACATTGTGGACCCTGTATTC (BamHI)<br>as: ACTATGGGTACCATTATGATGAGCGCTCCACAGCATCATCTAACAGTC (KpnI)	This study
36. pKNT25-*bcsE*^{FL}	MGSM^{1}… S^{523}-GTSNSMT-T25 ; 84.8 kDa	Vector MCS redesign: as in 32. *bcsE*^{FL} cloning:<br>s: CATATGGGATCCATGAGGGACATTGTGGACCCTGTATTC (BamHI)<br>as: ACTATGGGTACCTGATGAGCGCTCCACAGCATCATCTAACAGTC (KpnI)	This study
37. pUT18-*bcsE*^{FL}	MGSM^{1}… S^{523}-GTSNS-T18 ; 79.4 kDa	Vector MCS redesign: as in 33. *bcsE*^{FL} cloning: same as 36.	This study
38. pUT18C-*bcsE*^{FL}	T18-HCRSTGS-M^{1}… ; 80 kDa	Vector MCS redesign: as in 34. *bcsE*^{FL} cloning: same as 35.	This study
39. pKT25-*bcsE*$^{217-523}$	T25-GSTLEGS-A^{217}… ; 59.7 kDa	Vector MCS redesign: as in 31. *bcsE*$^{217-523}$ cloning:<br>s: ACCATGGGATCCGCGGAGATCCAACCACGCAGC (BamHI)<br>as: ACTATGGGTACCATTATGATGAGCGCTCCACAGCATCATCTAACAGTC (KpnI)	This study
40. pUT18C-*bcsE*$^{217-523}$	T18-HCRSTGS-A^{217}… ; 55.2 kDa	Vector MCS redesign: as in 34. *bcsE*$^{217-523}$ cloning: same as 39.	This study
41. pKT25-*s10*	T25-GSTLEGS-M^{1}… ; 36.7 kDa	Vector MCS redesign: as in 31.<br>s: CATATGGGATCCCAGAACCAAAGAATCCGTATCCGCCTGAAAG (BamHI)<br>as: ACTATGGGTACCATTAACCCAGGCTGATCTGCACGTCTAC (KpnI)	This study

42. pUT18C-*s10*	T18-HCRSTGS-Q^{2}…	same as in 34. (vector) and 41. (insert)	This study
43. pKT25-*nusB*	T25-GSTLEGS-M^{1}… ; 40.6 kDa	Vector MCS redesign: as in 31.<br>s: CATGTAGGATCCATGAAACCTGCTGCTCGTCGCC (BamHI)<br>as: ACT GTA GGT ACC ATCA CTT TTT GTT AGG GCG AAT CAC AGG TGC (KpnI)	This study
44. pRSFDuet1*-*s10*	S10: M^{1}GSQ^{2}… ; 12 kDa	Vector MCS1 redesign: as in 13. *s10* cloning in site 1:<br>s: CATATGGGATCCCAGAACCAAAGAATCCGTATCCGCCTGAAAG (BamHI)<br>as: CTATAGGCGGCCGCTTAACCCAGGCTGATCTGCACGTCTAC (NotI)	This study
45. pRSFDuet1*-*s10*$^{(site1)}$-*nusB*$^{(site2)}$	S10: as in 44.<br>NusB: MKLM^{1}… ; 16 kDa	Vector MCS1 redesign: as in 13. *s10* cloning in site 1: as in 44.<br>Vector MCS2 redesign:<br>s: GAATCCCTCGAGTCTGGTAAAGAAACCGCTGCTG (XhoI)<br>as: GGATCCAAGCTTCATATGTATATCTCCTTCTTATAC (HindIII)<br>*nusB* cloning in site 2:<br>s: GGATCCAAGCTTATGAAACCTGCTGCTCGTCGCC (HindIII)<br>as: GGATCCCTCGAGTCACTTTTTGTTAGGGCGAATCACAGGTGC (XhoI)	This study
46. pProExHTB-*nus*^{His}*B*	Nus^{His}B: MSYYHHHHHHDYDIPTTLEVLFQ-✂-GPMGSM^{1}… ; 19 kDa (with tag)	s: CATGTAGGATCCATGAAACCTGCTGCTCGTCGCC (BamHI)<br>as: ACT GTA GGT ACC ATCA CTT TTT GTT AGG GCG AAT CAC AGG TGC (KpnI)	This study
47. pRSFDuet1*-*bcsE*$^{1-217(site1)}$-*s10*$^{(site2)}$	BcsE$^{1-217}$: as in 14.<br>S10: M^{1}KLQ^{2}… ; 12 kDa	Vector MCS1 and MCS2 redesign: as in 45. *bcsE*$^{1-217}$ cloning in site 1: as in 14.<br>*s10* cloning in site 2:<br>s: GGATCCAAGCTTCAGAACCAAAGAATCCGTATCCGCCTGAAAG (HindIII)<br>as: GAATCCCTCGAGTTAACCCAGGCTGATCTGCACGTCTAC (XhoI)	This study
48. pProExHTB-*min*^{His}*DE*	Min^{His}D: MSYYHHHHHHDYDIPTTLEVLFQ-✂-GPMGSM^{1}…<br>MinE: full-length protein; did not co-purify with Min^{His}D from the cytosolic fraction	s: CATATGGGATCCATGGCACGCATTATTGTTGTTACTTCGGGC (BamHI)<br>as: CTAGAAGCGGCCGCTTATTTCAGCTCTTCTGCTTCCGGTAAGGTCAC. (NotI)	This study
