# Supplementary figures and images for: Structure and Multitasking of the c-di-GMP-Sensing Cellulose Secretion Regulator BcsE
Source: mBio. 2020 Aug 11;11(4):e01303-20. doi: 10.1128/mBio.01303-20 (PMC7439463; doi:10.1128/mBio.01303-20)

BcsF<sup>Enterobacteriales</sup>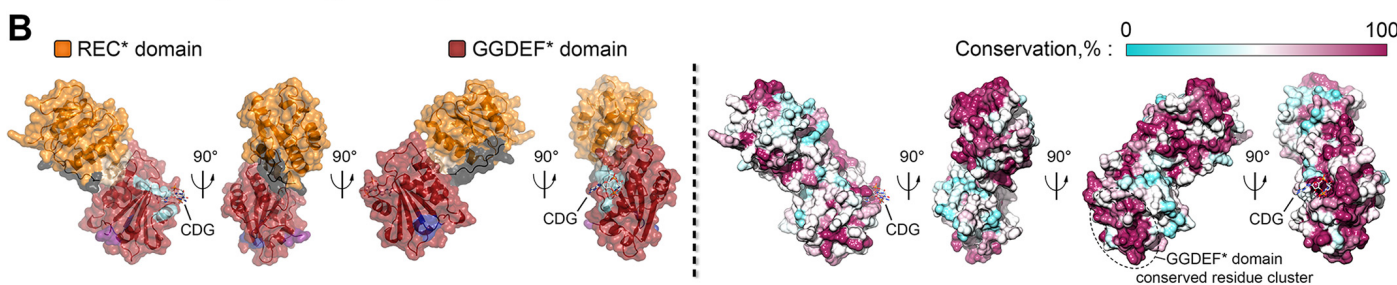

Supplementary Figure 1

Supplement: FIG S1 [file mBio.01303-20-sf001.pdf]
