# Supplementary material for: Structure and Multitasking of the c-di-GMP-Sensing Cellulose Secretion Regulator BcsE
Source: mBio. 2020 Aug 11;11(4):e01303-20. doi: 10.1128/mBio.01303-20 (PMC7439463; doi:10.1128/mBio.01303-20)
Supplement: FIG S2 [file mBio.01303-20-sf002.pdf]

A

```
Consensus   1 --------------------------------------MRD-IMAPSFSLGIRSLWDELRHMPAGGVWWVNXDRQEDAISLCNQTIAAQAENAKVALICMGEDPRKLLXLLPDXSXGPXKIPLFSXPNQEK  92
PP 2629     1 MTQLCVTHHFCRFFDMSHKASAEDVTHSAFKPFQFTFCRASVLTNGMQLLAISGLSVESSQLHQQGLYWLACDAAEDASLLCRQVVEGMPDNARAALIADAQAMDDVLGAL-DATRGPAELALYEANSQAT 130

Consensus  93 ALYFLXRDLLRSLDPENYLFILLCANNAWQNITXEELRXWLEKMNKWLRYHHCTLLVINPGNNNDKQRSQL----MSEYRSLFGLASLR--XQQDSHLYDXAWWCNEKGVSARQQLTLXHXEGGWQLADXE 217
PP_2629   131 --RYLVED-LPRLDARGRVLVLLAPAHAWADTE---VEHWCNALRQALLAEAALLLVVSGGAEHCLGRAPARFQPVPRWPG-AGVPRQRWGALPAAFLEQPAWQNRHPGHGADS----------------- 237
```

B

```
Consensus 133 LEKMNKWLRYHHCTLLVINPGNNNDKQRSQLMSEYRSLFGLASLRXQQDSHLYDXAWWCNEKGVSARQQLTLXHXEGGWQLADXEEQXTPXQPRSDEKRILSXVAVLEGAPPLSEHWQLFEXNEALFNEART 264
PP_2630     1 ----------------------------------------------------------------------------------------------------MLCLAQRPVLEGAPAFSEHWQVCESFEELGGKASR  35

Consensus 265 AQAATLIFSLTQNNQIEPLARQIHTLRRQRGSALKIVVREMTASLRATDERLLLACGANMXIPWNAPLSRCLTLIESXQGQQFSRHVPEDIDTLLSMTQPLKLRGYQPWDVFCQAVXNLMNNTLLPEDGKGV 396
PP_2630    36 AVSATVIFAMDGGQRLDSLARQLHHLRQSRGNALKLVVREMASTLRYQDEQLLLACGASQIVPFGASLSRFLTMVESIQGYVWRRHLPTDFDALLARLRPLAICGLVAPRAFADAVQSMWHGVR-NGEIVHQ 166
```

[redacted]
