# Supplementary material for: Structure and Multitasking of the c-di-GMP-Sensing Cellulose Secretion Regulator BcsE
Source: mBio. 2020 Aug 11;11(4):e01303-20. doi: 10.1128/mBio.01303-20 (PMC7439463; doi:10.1128/mBio.01303-20)
Supplement: TABLE S2 [file mBio.01303-20-st002.pdf]

Crystallographic data collection and refinement statistics	
	BcsE$^{217\text{-}523}$
Crystallized protein	SeMet
Data Collection	
Space group	P 41 21 2
Cell dimensions	
a, b, c (Å)	112.5, 112.5, 106.4
α, β, γ (deg)	90, 90, 90
Wavelength	0.9791
Resolution (Å)	49.7 – 2.2 (2.28 – 2.2)
R-merge	21.1% (257.6%)
R-meas	21.7% (264%)
R-pim	5.2% (63.3%)
Mean $I/\sigma(I)$	12.61 (1.1)
Completeness (%)	99.3% (96.8%)
Multiplicity	17.3
$CC_{1/2}$	99.9 (65.2)
Refinement	
Unique reflections	35 074
R-work	20.5%
R-free	23.8%
Number of non-hydrogen atoms	
Proteins	4 091
Ligands	58
B-factors	
Proteins	60.48
Ligands	50.51
R.m.s.d.	
Bond lengths (Å)	0.013
Bond angles (deg)	1.67
Ramachandran plot	
Favored (%)	96.9
Allowed (%)	2.9
Molprobity score	1.51
Crystallization condition ligand	c-di-GMP
Ligand in structure **	c-di-GMP
Protein : c-di-GMP ratio	2 : 1

** Statistics for the highest-resolution shell are shown in parentheses*
